# Comprehensive Analysis of Transcript Start Sites in Ly49 Genes Reveals an Unexpected Relationship with Gene Function and a Lack Of Upstream Promoters

**DOI:** 10.1371/journal.pone.0018475

**Published:** 2011-03-31

**Authors:** Frances Gays, Alan S. C. Koh, Katarzyna M. Mickiewicz, Jonathan G. Aust, Colin G. Brooks

**Affiliations:** The Institute of Cell and Molecular Biosciences, The Medical School, Newcastle, United Kingdom; University of Hawaii Manoa, United States of America

## Abstract

Comprehensive analysis of the transcription start sites of the *Ly49* genes of C57BL/6 mice using the oligo-capping 5′-RACE technique revealed that the genes encoding the “missing self” inhibitory receptors, Ly49A, C, G, and I, were transcribed from multiple broad regions in exon 1, in the intron1/exon2 region, and upstream of exon -1b. *Ly49E* was also transcribed in this manner, and uniquely showed a transcriptional shift from exon1 to exon 2 when NK cells were activated in vitro with IL2. Remarkably, a large proportion of *Ly49E* transcripts was then initiated from downstream of the translational start codon. By contrast, the genes encoding Ly49B and Q in myeloid cells, the activating Ly49D and H receptors in NK cells, and Ly49F in activated T cells, were predominantly transcribed from a conserved site in a pyrimidine-rich region upstream of exon 1. An ∼200 bp fragment from upstream of the *Ly49B* start site displayed tissue-specific promoter activity in dendritic cell lines, but the corresponding upstream fragments from all other *Ly49* genes lacked detectable tissue-specific promoter activity. In particular, none displayed any significant activity in a newly developed adult NK cell line that expressed multiple Ly49 receptors. Similarly, no promoter activity could be found in fragments upstream of intron1/exon2. Collectively, these findings reveal a previously unrecognized relationship between the pattern of transcription and the expression/function of Ly49 receptors, and indicate that transcription of the *Ly49* genes expressed in lymphoid cells is achieved in a manner that does not require classical upstream promoters.

## Introduction

The *Ly49* gene complex on mouse chromosome 6 encodes a family of closely related type II transmembrane proteins most of which have been shown to recognize MHC class I or class I-related ligands [1], [2]. In the C57BL/6 mouse strain there are probably 10 functional genes, but despite their close linkage and high degree of sequence similarity, they show quite different patterns of expression. The four genes that encode the inhibitory “missing self” receptors Ly49A, C, G, and I are expressed on NK cells, NKT cells, and activated T cells in an unusual stochastic manner creating a complex repertoire of cells expressing different permutations of these receptors in a pseudo-monoallelic manner [3], [4], [5]. Ly49F is found predominantly on a subpopulation of activated T cells [6], whilst Ly49E is expressed in the absence of other Ly49s on fetal NK cells, thymic NKT cells, various subpopulations of resting and activated γδT cells, and on activated (but not resting) mature NK cells [7], [8], [9]. Whether the expression of Ly49F is biallelic or monoallelic is unknown, but Ly49E appears to be expressed in a stochastic and predominantly monoallelic manner [10], [11]. Expression of the two genes encoding activating receptors, Ly49D and H, is strictly limited to NK cells, and has been reported to occur in a non-stochastic and biallelic manner [12], [13]. The remaining two Ly49s, Ly49B and Q are expressed on separate but partially overlapping myeloid cell populations in an apparently non-stochastic manner [14], [15].

Surface expression of Ly49 receptors correlates closely with expression of the corresponding mRNAs. This implies that expression is regulated predominantly at the transcriptional level and that sequence differences within the individual genes and their flanking regions are responsible for the different patterns of expression. A pre-requisite for understanding the expression of these genes, therefore, would be to establish the sites at which transcription is initiated and the locations of regulatory sequences. Previous studies have indicated considerable variability in the positions of transcription start sites (TSSs) both between and within individual *Ly49* genes [16], [17], [18]. However, it is not clear whether this variation is real or a consequence of RNA degradation or the premature termination of reverse transcription during the preparation of cDNA. Furthermore, only a few *Ly49* genes have been examined. Similarly, the analysis of regulatory sequences has been largely confined to the immediate upstream regions of two genes encoding inhibitory receptors expressed in NK cells, *Ly49A* and *Ly49I*, and has relied exclusively on in vitro promoter assays in transfected EL4 cells [17], [19], [20], [21]. In the case of *Ly49I*, such studies led to the identification of a potential repressor site upstream of the core promoter [20], but the existence of this site in other genes has not been examined. In the case of *Ly49A*, putative binding sites for the transcription factors AML1, TCF-1, and TBP upstream of exon 1 were suggested to control expression [17], [19], but puzzlingly these sites are not well conserved even amongst the four inhibitory genes, and the promiscuity of transcription factor binding sites has led to the validity of this general approach being questioned [22]. By contrast, the chance cloning of a rare *Ly49G* transcript led to the identification of a distal upstream element, Pro1, that is highly conserved amongst the inhibitory genes and which has been reported to act as a bidirectional promoter in immature, but not mature, NK cells [23], [24].

In the present study we set out to unambiguously establish the nature of transcriptional initiation in each of the functional *Ly49* genes of C57 mice using the “oligo-capping” RACE technique [25] in which an RNA oligonucleotide is ligated to the 5′ end of mRNA molecules in a reaction that is dependent on the presence of a cap structure, thereby ensuring that only full length undegraded RNA molecules and full length cDNA copies that extend to the 5′ end of the mRNA are captured. The results revealed for the first time distinct variations in transcriptional patterns that correlate with *Ly49* gene expression patterns and function. Surprisingly, only in the case of the *Ly49B* gene did the regions upstream of transcriptional initiation sites display clear tissue specific promoter activity.

## Materials and Methods

### Ethics statement

All animal work was approved by the UK Home Office under licence 60-3379.

### Cells and RNA

Spleen, bone-marrow, and peritoneal cells were prepared using standard methods from normal C57BL/6 mice, or from RAG-ko mice on a C57BL/6 background kindly provided by Dr. B. Seddon, National Institute of Medical Research, London, the latter serving as a source of fresh ex vivo NK cells. Cultured NK cells, comprising >98% NK1.1^+^CD3^−^ cells, were obtained by sorting fresh C57BL/6 spleen cells that displayed high levels of staining with PE-Cy7 anti-NK1.1 (BD Biosciences) using a FACSDiva instrument (BD Biosciences), and growing these in DMEM containing 10% FBS and 350 ng/ml human rIL2 for ∼3 weeks [26]. The D^−^ NK cell line arose from a similar experiment in which, after 5 days culture, cells were stained with the 2D9 anti-NKRP1D mAb [27], sorted for NKRP1D^−^ cells, and returned to culture. After ∼3 months, outgrowth of cells having an NK1.1^+^CD3^−^ phenotype occurred, and these could be cloned and maintained indefinitely in the above medium. Immature NK cells, comprising >95% NK1.1^+^CD3^−^ cells, were generated from day 14 fetal thymocytes as described previously [28], [29]. The I2/22 immature NK cell line was obtained by extended culture and subsequent cloning of such cells. The LNK immature NK cell line [23], [30] was obtained from Dr. S. Anderson, NCI- Frederick, MD. Both of these lines were grown in DMEM containing 10% FBS and 3.5 ng/ml human rIL2. Cultured bone-marrow derived macrophages and dendritic cells were also generated as described previously [15]. Mouse tumour cell lines were grown in continuous culture in DMEM with 5% FBS.

### Oligo-capping RACE

**T**he GeneRacer kit from Invitrogen was used exactly as described by the manufacturer. Briefly, RNA prepared using RNA Bee (Biogenesis) according to the manufacturer's instructions was treated with calf intestinal phosphatase to remove 5′ phosphate groups, and then with tobacco acid pyrophosphatase to remove the 5′ cap structure from intact mRNA molecules, leaving an exposed 5′ phosphate group that provided the substrate for RNA ligase mediated ligation of a GeneRacer RNA oligonucleotide. cDNA copies were generated using random primers so as to avoid bias towards the 3′ ends, followed by PCR amplification using the GeneRacer forward primer and an appropriate gene specific reverse primer. This strategy ensured that only transcripts that possessed a 5′ cap structure and which were reverse transcribed to the 5′ end of the ligated oligonucleotide would generate PCR products [25]. Second round PCR was performed using the GeneRacer nested primer and an internal gene specific reverse primer. The sequences and locations of the main primers used are shown in Table S1 and Figure S1. The products were then cloned into pCR4-TOPO-TA (Invitrogen) and sequenced. To avoid biases, multiple independent RACE-PCR reactions were performed with multiple independent RNA samples and with at least two combinations of reverse primers for each gene. In addition, no more than two sequences that had the same 5′ start point were included from the same reaction in the final dataset. Statistical analysis was by Fisher's exact test.

### Conventional RT-PCR

RNA prepared as above was reverse transcribed using an oligo(dT) primer and Moloney murine leukaemia virus reverse transcriptase (Promega) according to the manufacturer's instructions. PCR was performed by adding cDNA prepared from equal numbers of cells to PCR mixtures containing 200 µM dNTPs, 2 mM MgCl_2_, 0.1 µM forward and reverse primers, and 20 U/ml Taq polymerase (Bioline) in NH_4_ buffer. Reactions were run for 40 cycles at 95°C for 1 min, 58°C for 1 min, and 72°C for 1 min, then examined on agarose gels containing ethidium bromide. The sequences of the primers used are given in Table S1. In appropriate cases the amplimers were cloned into pCR4 and sequenced.

### Immunofluorescence

Cells were stained with the following mAbs, some kindly provided by the investigators shown, and analysed by flow cytometry: JR9 anti-Ly49A (Dr. J. Roland, Pasteur Institute, Paris, France), SED85 anti-Ly49D (Dr. D. Raulet, University of California), CM4 anti-Ly49E [9], HBF anti-Ly49F (BD Bioscience), 4LO3311 anti-Ly49C (Dr. S. Lemieux, University of Quebec, Canada), 4D11 anti-Ly49G (Dr. L. Mason, NIH, Bethesda, MD), 3D10 anti-Ly49H (Dr. H. Smith and W. Yokoyama, Washington University School of Medicine, St. Louis, MO), YLI-90 anti-Ly49I (BD Biosciences), PE-Cy7 anti-NK1.1 (BD Biosciences), and eFluor660 anti-CD3 (eBioscience).

### Constructs and luciferase reporter assays

The desired segments from *Ly49* genes were amplified from appropriate BACs from the RP23 and RP24 mouse and CH230 rat libraries (BACPAC Resources Center, CHORI) using primers containing 5′ EcoRV and HindIII sites and KOD HiFi polymerase (Novagen). The HSV promoter was amplified from the pNS vector [31] in the same manner. The mutant B-448-Inr-GG fragment was prepared by direct gene synthesis (MWG, Ebersberg, Germany). The products were digested with EcoRV and HindIII and cloned into the EcoRV and HindIII sites of the promoterless luciferase reporter vector pGL4 (Promega). The full sequences of all of the fragments tested in this study are given in **Table S2**. Plasmids were purified using Genelute kits (Sigma), and transfected into cells using DEAE-dextran. Briefly, cells were washed in Ca-free PBS then resuspended in AIMV medium (Invitrogen). Aliquots of 1.5 million cells were incubated with 1 µg/ml DNA and 100 µg/ml DEAE-dextran for 30 min at room temperature in a volume of 2 ml AIMV, then spun down, resuspended in DMEM/HEPES (Sigma D6171) containing 10% FBS, and duplicate 1 ml aliquots added to wells of 24 well plates. In the case of the D^−^ NK cells this medium also contained 350 ng/ml human rIL2. One day later, non-adherent cells were resuspended and transferred to microfuge tubes, spun down, and supernatant discarded. Meanwhile, 100 µl of Glow Lysis Buffer (Promega) was added to the remaining adherent cells in each well and, after 10 min incubation at room temperature with occasional mixing, the lysed cells were resuspended and transferred to the cell pellets in microfuge tubes. Following mixing, 30 µl of lysate was mixed with 10 µl of Bright-Glo reagent (Promega) in the wells of luminescence plates (Thermo 7705). These were loaded into a Thermo Varioskan luminometer, and scanned after 10 min dark adaptation. In all experiments empty pGL4 was used as a negative control, and pGL4 containing the HSV promoter as positive control. The luciferase activity of all samples was calculated as a percentage of that of the positive control HSV promoter plasmid. In a typical experiment, the control pGL4-HSV promoter construct had reporter activity >30 fold higher than empty pGL4 vector. The data shown represents the mean values obtained in at least three independent experiments with each construct. Statistical analysis was by paired t-test.

## Results

### Transcriptional start sites of C57 *Ly49* genes

**Figure 1:**
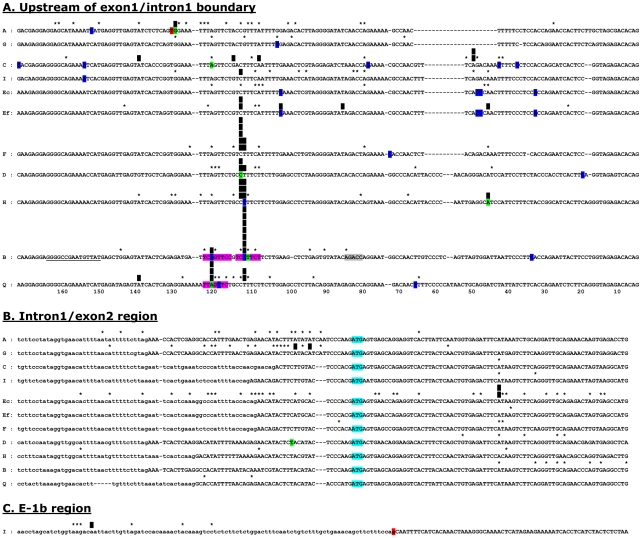
Transcriptional start sites in *Ly49* genes. The start sites of cap-trapper 5′RACE cDNAs are plotted onto gene sequence alignments of the region upstream of the exon1-intron1 boundary (A), the region surrounding the intron1-exon2 boundary (B), and exon-1b (C). Exonic regions are shown in upper case letters, intronic regions in lower case letters. In the case of the *Ly49I* exon-1b region, the distinction is artificial as the intronic region ends with a defective splice signal (highlighted in red). Black vertical bars mark sites at which >10% of all transcripts for a given gene originated, the number of bars indicating the actual percentage in units of 10% (eg. 3 bars = 30–40% of all transcripts). Asterisks show other TSSs in these regions. A small number (<5%) of TSSs mapped outside of these egions (see text) and are therefore not shown on the diagram, but are included in the analysis in Table 1. Bases highlighted in blue or green are the TSSs of cDNAs deposited in Genbank, those in green being from the Riken CAP-trapper high efficiency cDNA cloning project. The red T in *Ly49A* is the TSS for *Ly49A* in EL4 cells determined by Kubo et al [16] using primer extension and nuclease protection. Regions highlighted in purple in *Ly49B* and *Ly49Q* correspond to canonical Inr sequences, the grey region in *Ly49B* corresponding to a perfectly positioned canonical DRE sequence, and the underlined bases in *Ly49B* corresponding to GC and TA rich regions with similarity to BRE and TATA sequences [46]. For 9 of the 10 genes, the general disposition of TSSs in fresh cells was not different to that in cultured cells (see Table 1), and the data has therefore been combined. However, for *Ly49E* the results obtained from fresh cells (Ef) and cultured cells (Ec) were markedly different and are therefore plotted separately.

Oligo-capping 5′RACE analysis of C57 *Ly49* transcripts revealed striking differences in the nature and position of their TSSs that showed a remarkable correlation with the function and expression pattern of the genes. A graphical representation of the data is provided in Figure 1, with a full numerical analysis in Table 1. The start sites of the two *Ly49* genes expressed in myeloid cells, *Ly49B* and *Ly49Q*, were highly restricted, the great majority of transcripts originating from exon 1, and predominantly from a highly favoured region within exon 1. In the case of *Ly49B*, 60% of all transcripts initiated from a single A residue 105 bases upstream of the exon1/intron1 boundary, and 11% initiated at an A residue 9 bases further upstream. In *Ly49Q*, 27% and 30% of all transcripts initiated at the exactly homologous positions. The dominance of these start sites in *Ly49B* and *Ly49Q* was observed in all myeloid cell-containing populations examined including fresh spleen cells, fresh peritoneal cells, fresh bone marrow cells, cultured bone marrow-derived macrophages, cultured bone marrow-derived dendritic cells, and the myeloid tumour cell line RAW264. The genes encoding activating Ly49 receptors in C57 NK cells, *Ly49D* and *Ly49H*, also used this same homologous site in exon 1 as the major TSS, 64% of all *Ly49H* transcripts and 43% of all *Ly49D* transcripts beginning at the base corresponding to that which formed the dominant TSS in *Ly49B*, or at the preceding base. In addition, 70% of all transcripts from the *Ly49F* gene initiated at the corresponding homologous position, which was also favoured, but to a lesser extent, during in vivo transcription of *Ly49E*.

**Table 1:** Summary of all RACE data^1^.

		Total	% E-1b	% E1	% I1/E2	% E2 No ATG	% >E2	% No ATG
**Ly49A**	Cultured	38	0	58	32	5	5	11
	Fresh	29	0	72	28	0	0	0
	**Total**	**67**	**0**	**64**	**30**	**3**	**3**	**6**
**Ly49B**	Cultured	58	0	88	0	12	0	12
	Fresh	56	0	84	0	16	0	16
	**Total**	**114**	**0**	**86**	**0**	**14**	**0**	**14**
**Ly49C**	Cultured	19	0	89	0	11	0	11
	Fresh	10	0	90	0	0	10	10
	**Total**	**29**	**0**	**90**	**0**	**7**	**3**	**10**
**Ly49D**	Cultured	30	0	67	0	23	10	33
	Fresh	19	0	68	0	21	11	32
	**Total**	**49**	**0**	**67**	**0**	**22**	**10**	**33**
**Ly49E**	Cultured fetal	62	0	16	31	53	0	53
	Cultured adult	69	0	22	26	51	1	52
	**All cultured**	**131**	**0**	**19**	**28**	**52**	**1**	**53**
	Fresh	17	0	94	0	6	0	6
	**All fresh**	**17**	**0**	**94**	**0**	**6**	**0**	**6**
**Ly49F**	Cultured	15	0	93	7	0	0	0
	Fresh	22	0	82	0	14	5	18
	**Total**	**37**	**0**	**86**	**3**	**8**	**3**	**11**
**Ly49G**	Cultured	39	1	13	72	3	13	15
	Fresh	20	0	10	50	0	40	40
	**Total**	**59**	**1**	**12**	**64**	**2**	**22**	**24**
**Ly49H**	Cultured	37	0	95	3	0	3	3
	Fresh	32	0	84	6	3	6	9
	**Total**	**69**	**0**	**90**	**4**	**1**	**4**	**6**
**Ly49I**	Cultured	25	28	60	4	8	0	8
	Fresh	8	75	25	0	0	0	0
	**Total**	**33**	**39**	**52**	**3**	**6**	**0**	**6**
**Ly49Q**	Cultured	38	0	97	3	0	0	0
	Fresh	23	0	96	4	0	0	0
	**Total**	**61**	**0**	**97**	**3**	**0**	**0**	**0**

1 For each *Ly49* gene the data shows the total number of RACE cDNA clones obtained from either cultured or fresh cells, and the percentage of these that originated in exon -1b (E-1b), exon 1 (E1), the region around the intron 1/exon 2 boundary upstream of the ATG start codon (I1/E2), in exon 2 downstream of the ATG start codon (E2 No ATG), or downstream of exon 2 (>E2), together with the total percentage of transcripts that initiated downstream of the normal translational start codon in exon 2 (% No ATG).

By contrast, amongst the four genes known to encode inhibitory receptors in NK cells, *Ly49A*, *C*, *G*, and *I*, no consistent dominant TSS was found. Instead, transcripts were initiated over a broad region not only within exon 1 but at other sites. In particular, 64% of *Ly49G* and 30% of *Ly49A* transcripts initiated in a broad region around the intron1/exon2 boundary. Although other *Ly49* genes also initiated transcripts from this region the frequency was much lower. For example, 3/62 *Ly49H* transcripts began in this region compared to 20/67 *Ly49A* transcripts (P = 0.002). *Ly49C* initiation was largely confined to exon 1, but surprisingly a large proportion (39%) of *Ly49I* transcripts initiated from an LTR-like repeat sequence just upstream of exon -1b [23]. A single *Ly49G* transcript was also found to start in this region. Alignment of the regions upstream of exon -1b revealed no obvious reason why *Ly49I* transcripts should so frequently originate from this site, the *Ly49I* sequence being very similar to that of *Ly49C*, *E*, and *F* (not shown).

Uniquely, in the case of *Ly49E* a striking difference was found between fresh cells and cultured cells: in fresh cells 94% of transcripts initiated in exon 1 and 0% at the intron1/exon2 boundary, but in cultured cells only 19% of transcripts initiated in exon 1 and 28% initiated around the intron1/exon2 boundary (P = 1.3×10^−9^). In addition, and unexpectedly, 52% of all *Ly49E* transcripts in cultured NK cells initiated downstream of the ATG translational start codon with nearly half of these initiating at a single A residue 40 bases downstream of the start codon. No difference in the distribution of *Ly49E* initiation sites was found between immature NK cells cultured from fetal thymus and mature NK cells cultured from adult spleen. Most other genes, including those with dominant sites in exon 1, also initiated transcripts from downstream of the ATG start codon in exon 2 but at lower frequencies than *Ly49E*. *Ly49* transcripts initiating outside of the regions shown in **Figure 1** were also found occasionally, including ones from further upstream in exon 1, and in exons and introns downstream of exon 2. The frequency of such transcripts is almost certainly underestimated due to biases against long cDNAs and the inability to detect transcripts initiating downstream of the reverse primers used.

### Analysis of sequence upstream of exon 1

**Figure 2:**
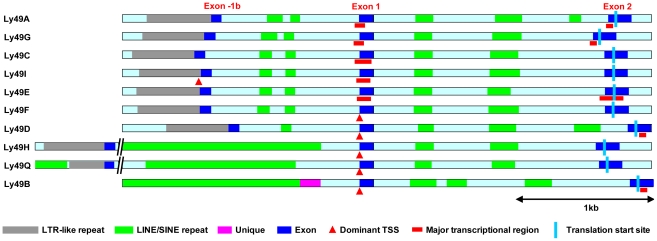
Structure of the upstream region of *Ly49* genes. The diagram shows the ∼3.5–6 kb region that extends upstream from exon 2 to just beyond the LTR-like repeat sequence that precedes exon -1b (except for *Ly49B* that lacks a recognizable exon -1b). Exons are shown in dark blue, homologous intronic regions in light blue, LINE/SINE repeats in green, LTR-like repeats in grey, and unique sequence in purple. Dominant TSSs are shown by red triangles, broad transcriptional initiation regions by red bars, and the translational start site by a pale blue line.

The comparative structure of the 5′ ends of the functional Ly49 genes in C57BL/6 mice is shown in Figure 2. All Ly49 genes show a high degree of organizational and sequence similarity from the start of exon 1 to downstream of exon 2. Ly49A, G, C, I, E, F, and D also show a high degree of similarity extending >2 kb upstream of exon 1 except that *Ly49D* has lost an ∼350 bp region from just downstream of exon -1b. The remaining three genes are strikingly different in this region. In the *Ly49B* gene only the first ∼290 bp upstream of exon 1 shows significant homology to the consensus *Ly49* sequence. No other sequence in the region extending at least 25 kb upstream of exon 1 in the *Ly49B* gene displays any significant homology to sequences found in the upstream region of other *Ly49* genes, except for repetitive elements. Most of the immediate upstream region in the *Ly49B* gene is occupied by a large block of LINE repeat sequence as noted previously [15]. Interestingly, immediately preceding this is a unique ∼120 bp sequence that has no homologue in any other Ly49 gene or anywhere else in the mouse genome. An homologous sequence is found in a similar position in the rat orthologue of *Ly49B*, *Ly49i8* (not shown), but not in any other rat *Ly49* gene, suggesting that this sequence may play some role in controlling the unique pattern of expression of *Ly49B*.

The *Ly49Q* and *Ly49H* genes also possess large LINE sequences in this region, but in contrast to the *Ly49B* gene most of the conserved upstream sequence found in the *Ly49A*, *G*, *C*, *I*, *E*, *F*, and *D* genes is still present, albeit displaced several kb upstream [not shown]. In the *Ly49H* gene the LINE sequence has been inserted just 280 bp upstream of the dominant TSS, and just 385 bp upstream of the exon1/intron1 boundary. Thus, the only uninterrupted upstream sequence shared by all C57BL/6 *Ly49* genes is the first ∼280 bp upstream of exon 1. According to conventional gene expression models this region would be expected to contain the core promoter elements required to drive *Ly49* gene expression.

### The upstream regions of most *Ly49* genes have little or no promoter activity in EL4 cells

**Figure 3:**
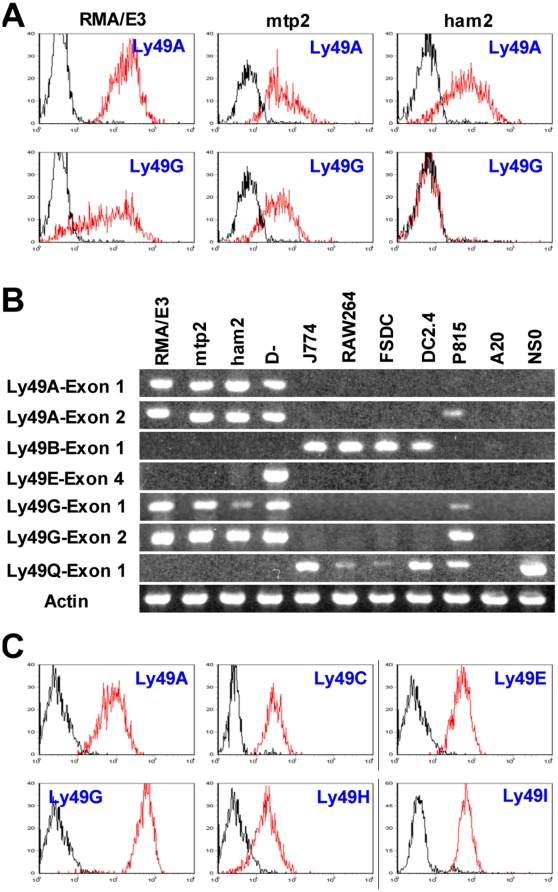
Expression of *Ly49* genes in various cells. A. Different sublines of RMA EL4 cells were stained with JR9 anti-Ly49A and 4D11 anti-Ly49G mAbs (red lines) or medium (black lines) followed by AF647-conjugated secondary antibody. B. RT-PCR analysis of *Ly49* transcripts in various cell lines. cDNA prepared from equal numbers of cells was amplified using forward primers located in exon 1, exon 2, or exon 4 together with appropriate reverse primers that in combination gave specific amplification of the relevant *Ly49*. The identity of the *Ly49A* exon 1 and *Ly49G* exon 1 amplimers in RMA/E3 cells was confirmed by cloning and sequencing, as was the unexpected presence of *Ly49A*, *G*, and *Q* transcripts in P815 cells, and of *Ly49Q* transcripts in NS0 cells. C. D^−^ NK cells were stained with mAbs against the Ly49s shown (red lines) or with medium (black lines).

To test this hypothesis, fragments from this region were inserted upstream of the luciferase gene in the promoterless pGL4 vector and transfected into EL4, a T cell lymphoma line that frequently but not always expresses Ly49A and Ly49G [32], and which has been used in all previous studies of *Ly49* gene promoters [17], [19], [20], [21]. The EL4 subline we used in the initial studies was RMA/E3 that expresses both Ly49A and Ly49G at the cell surface (Figure 3A, left panels) and at the transcriptional level (Figure 3B, lane 1).

**Figure 4:**
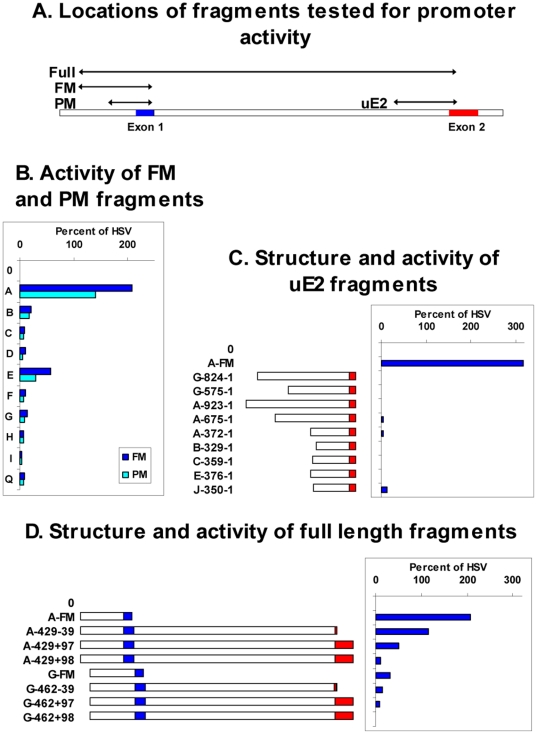
Promoter activity of *Ly49* gene fragments in EL4 cells. A. Schematic diagram of the gene fragments tested for promoter activity, namely the PM and FM fragments from the upstream region, the uE2 fragments from the region upstream of exon 2, and the full length ∼2.3 kb fragments that run from the start of the FM fragments to various positions around the start of exon 2. B–D. The RMA/E3 subline of EL4 cells was transfected with pGL4 plasmids containing no insert (0), the HSV promoter, or various *Ly49* gene fragments, as follows: B. FM (dark blue bars) and PM (light blue bars) fragments from the upstream region of the *Ly49* genes shown. C. The *Ly49A* FM fragment or fragments of various length extending upstream from the translational start codon in exon 2 of various *Ly49* genes. Numbers show the distance of the start and end of each fragment from the start codon, and the diagrams provide a graphical representation of the fragments with intron 1 unshaded and exon 2 in red. D. The FM fragments from the *Ly49A* and *Ly49G* genes, or “full length” fragments of various length beginning at the same upstream position as the FM fragments and ending at various positions in exon 2. Numbers show the distance of the start point from the exon1/intron1 boundary, and the distance of the end point from the translational start codon, a negative value meaning upstream and a positive value meaning downstream of the start codon. In the +97 constructs the *Ly49* start codon would be in frame with the luciferase start codon, in the +98 constructs it would be out of frame. The diagrams show a graphical representation of the fragments with exon 1 in blue, intron 1 unshaded, and exon 2 in red. In all cases, luciferase activity is expressed as a percentage of that observed with the HSV promoter.

The positions of the fragments tested are illustrated in Figure 4A. We initially examined the FM fragments, which in most cases began ∼430 bp upstream of the exon 1/intron 1 boundary and ended 12 bp upstream of this boundary, these sites being chosen because they corresponded with the Fnu4HI and MspI sites used in a previous study of the *Ly49I* promoter [20]. In the case of *Ly49H*, the FM product began shortly before the LINE sequence, 375 bp upstream of the exon1/intron1 boundary. Only the *Ly49A* fragment displayed substantive promoter activity, typically ∼100 fold higher than empty control vector and ∼2 fold higher than the control HSV promoter (Figure 4B, dark blue bars). By comparison, the FM fragments from most other genes had little or no activity, except that from *Ly49E* which displayed moderate activity.

The low activity of the *Ly49I* FM fragment agrees with previous results obtained by Gosselin et al. [20]. However, these workers reported that a truncated fragment of the *Ly49I* gene, beginning at a PstI site ∼270 bp upstream of the exon1/intron1 boundary had significant promoter activity leading to the suggestion that the distal part of the *Ly49I* FM fragment contained a repressor site. To investigate whether the presence of such a repressor site explained the lack of expression of other Ly49 genes, we prepared a series of pGL4 constructs containing the shorter upstream fragments equivalent to the PstI-MspI fragment of Ly49I (PM fragments, see Figure 4A). However, none of these ∼250 bp PM fragments, including that prepared from Ly49I, showed increased promoter activity compared to the longer FM fragment (Figure 4B, light blue bars).

### The relationship between promoter activity and the expression of endogenous *Ly49* genes

The high promoter activity of the *Ly49A* FM and PM upstream fragments in RMA/E3 cells correlates with the expression of the endogenous *Ly49A* gene in these cells. Similarly, the negligible promoter activity of most other *Ly49* upstream fragments in these cells correlates with the lack of expression of the corresponding endogenous genes at the protein level (data not shown) and at the RNA level (as illustrated by the RT-PCR data for *Ly49B* and *Ly49Q* in Figure 3B, lane 1). However, this correlation breaks down for the *Ly49G* gene, whose upstream fragment displays negligible promoter activity in RMA/E3 cells despite strong expression of the endogenous gene at the cell surface (Figure 3A, lower left panel) and at the RNA level (Figure 3B, lane 1).

This discrepancy might be explained if, as in NK cells, transcription of the *Ly49G* gene in RMA/E3 cells is initiated mainly at the intron1/exon2 boundary rather than upstream of exon 1. However, RT-PCR analysis using a forward primer site in exon 1 showed that exon 1-containing Ly49G transcripts could be readily detected in RMA/E3 cells (Fig 3B lane 1), in line with a previous RACE analysis that showed that most Ly49G transcripts in these cells originated from exon 1 [33]. Furthermore, fragments extending ∼500 bp and ∼800 bp upstream from just before the start codon in exon 2 of Ly49G (uE2 fragments, see Figure 4A) lacked detectable promoter activity in RMA/E3 cells (Figure 4C). Similar fragments prepared from other genes, including *Ly49A* and *Ly49E*, both of which like *Ly49G* frequently initiate transcription in NK cells at the intron1/exon2 boundary, were devoid of promoter activity, apart from some very low activity found in a fragment from the *Ly49J* gene (Figure 4C) that had previously been reported to display weak promoter activity [21]. In a further effort to identify promoter activity in the 5′ end of the *Ly49G* gene we tested “Full length” ∼2 kb constructs that began with the region upstream of exon 1, corresponding to the site of strong promoter activity in the *Ly49A* gene, and ended at various positions in exon 2 (see Figure 4A). None of these fragments had substantive promoter activity (Figure 4D). By contrast, a similar fragment from the *Ly49A* gene that ended 39 bp upstream of the start codon (A-429-39) was as active as the control HSV promoter, and an *Ly49A* fragment that ended 97 bp downstream of the start codon (A-429+97) retained significant activity despite the fact that such a construct would potentially add 44 amino acids to the N-terminus of the luciferase protein (Figure 4D). Only when one additional base was added (+98 construct), thereby causing a frame shift, was the promoter activity of *Ly49A* fragments lost.

**Figure 5:**
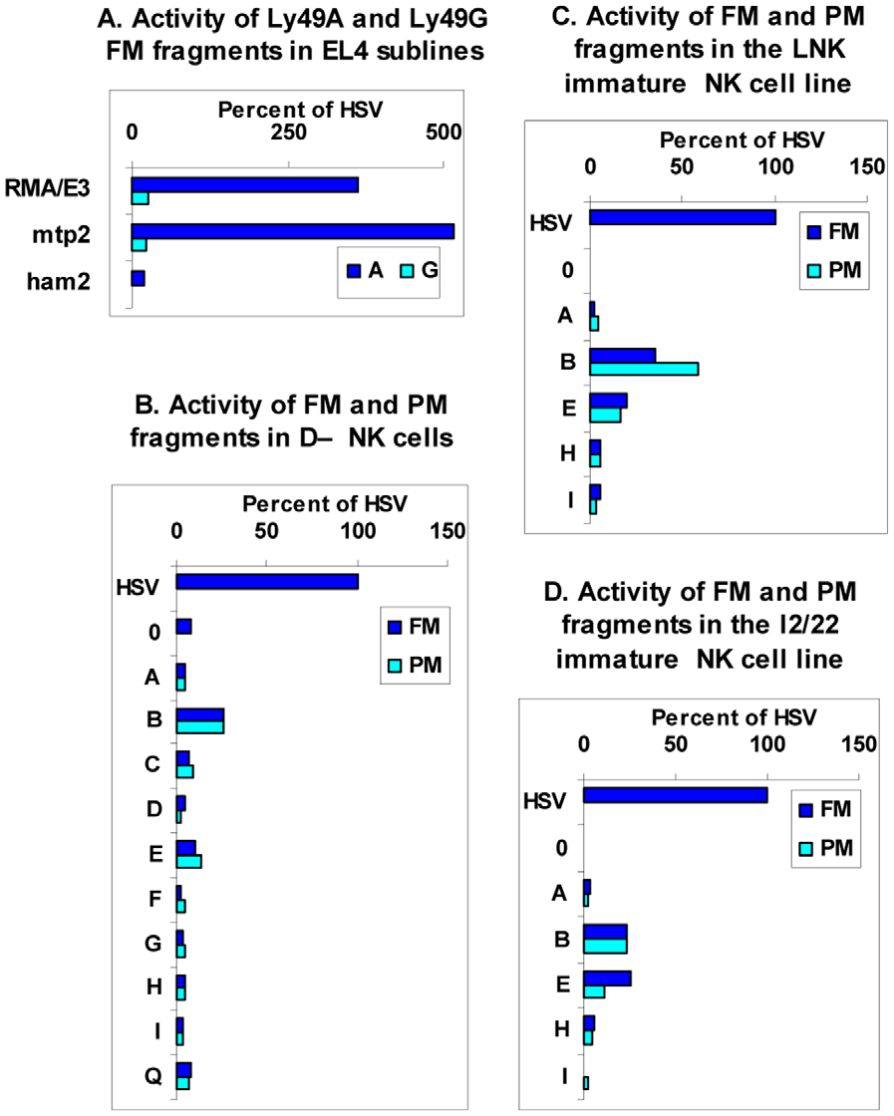
Lack of correlation between promoter activity and endogenous gene expression. A. The RMA EL4 sublines shown were transfected with pGL4 plasmids containing no insert (not shown), the HSV promoter, or the FM fragments from upstream of the *Ly49A* (dark blue bars) and *Ly49G* (light blue bars) genes. B–D. The mature NK cell line D^−^ (B), and the immature NK cell lines LNK (C) and I2/22 (D) were transfected with pGL4 plasmids containing no insert (0), or the FM (dark blue bars) and PM (light blue bars) fragments from upstream of the *Ly49* genes shown. In all cases, luciferase activity is expressed as a percentage of that observed with the HSV promoter.

To explore more fully the relationship between the promoter activity of upstream gene fragments and the expression of endogenous genes, we examined other sublines of RMA EL4 cells [32]. The *Ly49A* FM upstream fragment displayed strong promoter activity in the mtp2 subline [34] but only weak activity in the in the ham2 subline [35] (Figure 5A, dark blue bars) despite the fact that ham2 cells clearly expressed Ly49A at the cell surface (Figure 3A, upper right panel) and were actively transcribing the endogenous Ly49A gene (Figure 3B, lane 3). Similarly, the Ly49G FM fragment had negligible promoter activity in all three sublines (Figure 5A, light blue bars) despite the expression of Ly49G at the protein and/or RNA levels in all these cells (Figure 3A and 3B). Exactly parallel results were obtained with *Ly49A* and *Ly49G* PM fragments (data not shown).

It could be argued that because EL4 is a T cell line that has been grown in culture for more than 50 years, it is an inappropriate vehicle in which to analyse the physiological expression of Ly49 receptors in NK cells. During the course of this study we succeeded in deriving a stable long-term NK1.1^+^CD3^−^ NK cell line, D^−^, that uniformly expressed the Ly49 receptors A, C, E, G, H, and I as shown by RT-PCR analysis (Figure 3B, lane 4) and cell surface staining (Figure 3C). To our knowledge this is the first NK cell line to have been obtained from normal adult NK cells. Importantly, the FM and PM fragments from most Ly49 genes including Ly49A displayed no detectable promoter activity in D− NK cells (Figure 5B). The only fragments that displayed measurable, albeit low, activity were the FM and PM fragments from the Ly49B and Ly49E genes. Similarly, the various uE2 fragments from the intron1/exon2 boundary described above were completely inactive in D^−^ NK cells (not shown). Finally, to test the possibility that promoter activity in the upstream region of *Ly49* genes might be detectable in immature NK cells we transfected the FM and PM fragments of various *Ly49* genes into the immature NK cell lines LNK and I2/22. However, the pattern of results was similar to that obtained with D^−^ cells, most upstream fragments having little or no detectable promoter activity (Figure 5C and Figure 5D).

### Promoter activity of *Ly49B* and *Ly49Q* upstream regions

**Figure 6:**
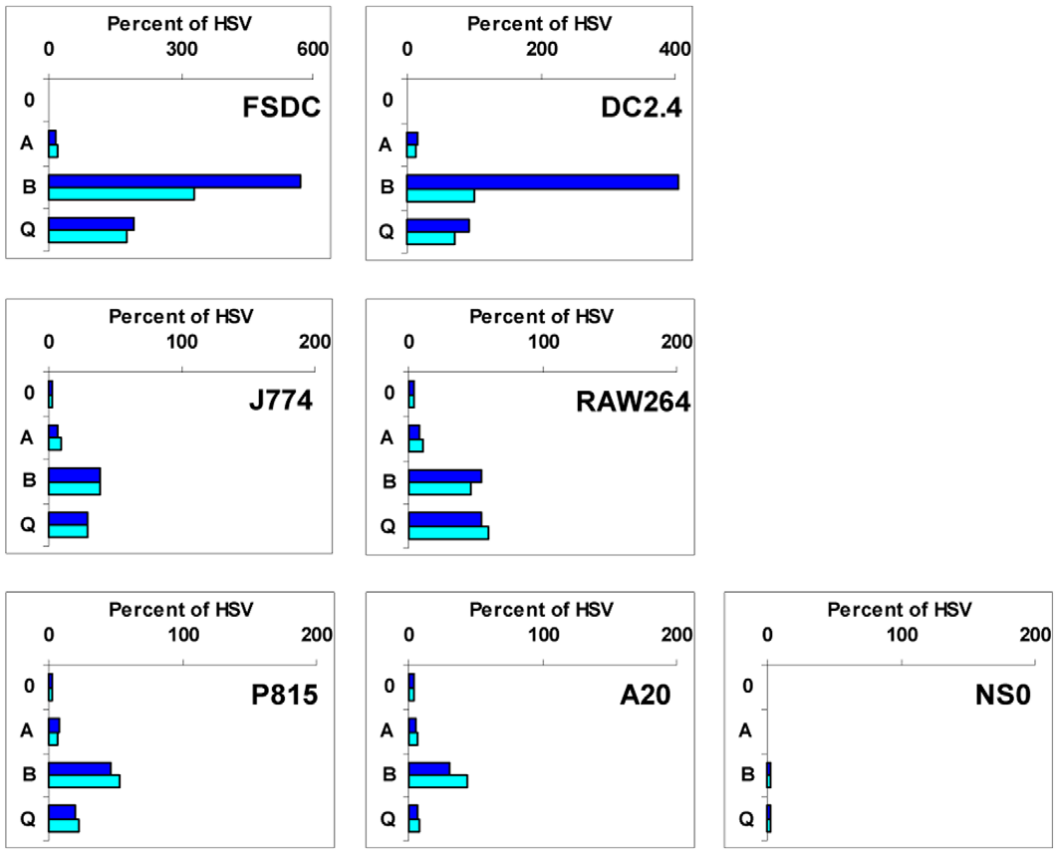
Analysis of promoter activity in the upstream region of the *Ly49B* and *Ly49Q* genes. pGL4 plasmids containing no insert (0), the HSV promoter, or the FM (dark blue bars) and PM (light blue bars) fragments from the upstream regions of the *Ly49A*, *B*, and *Q* genes were transfected into the DC lines FSDC and DC2.4, the macrophage lines J774 and RAW264, the mastocytoma P815, and the B cell lines A20 and NS0. Luciferase activity was calculated as a percentage of that observed with the HSV promoter. Note the different scale used for the DC lines.

Previous studies have shown that Ly49B and Ly49Q are mainly expressed in myeloid cells [14], [15], and this was confirmed in the present study by RT-PCR analysis which showed the presence of *Ly49B* and *Ly*49Q transcripts in the macrophage lines J774 and RAW264, and in the dendritic cell lines FSDC [36] and DC2.4 [37] (Figure 3B). To determine whether the upstream regions of the Ly49B and Ly*49Q* genes have selective promoter activity in such cells, appropriate constructs were transfected into these myeloid cell lines and into a number of non-myeloid cell lines. *Ly49A* FM and PM upstream fragments were devoid of activity in all of these cell lines (Figure 6). By contrast, Ly49Q FM and PM fragments displayed activity comparable to that of the positive control HSV promoter in the FSDC and DC2.4 dendritic cell lines. They also displayed moderate activity in the J774 and RAW264 macrophage lines and in the P815 mastocytoma line that like FSDC and DC2.4 express *Ly49Q* transcripts, but displayed negligible activity in the A20 and NS0 B cell lines despite the fact that NS0 cells were unexpectedly found to express high levels of endogenous *Ly49Q* transcripts (Figure 3B). Thus, although the upstream region of *Ly49Q* contains promoter activity that can be detected in various cell lines, this activity does not correlate precisely with the expression of the endogenous *Ly49Q* genes.

**Figure 7:**
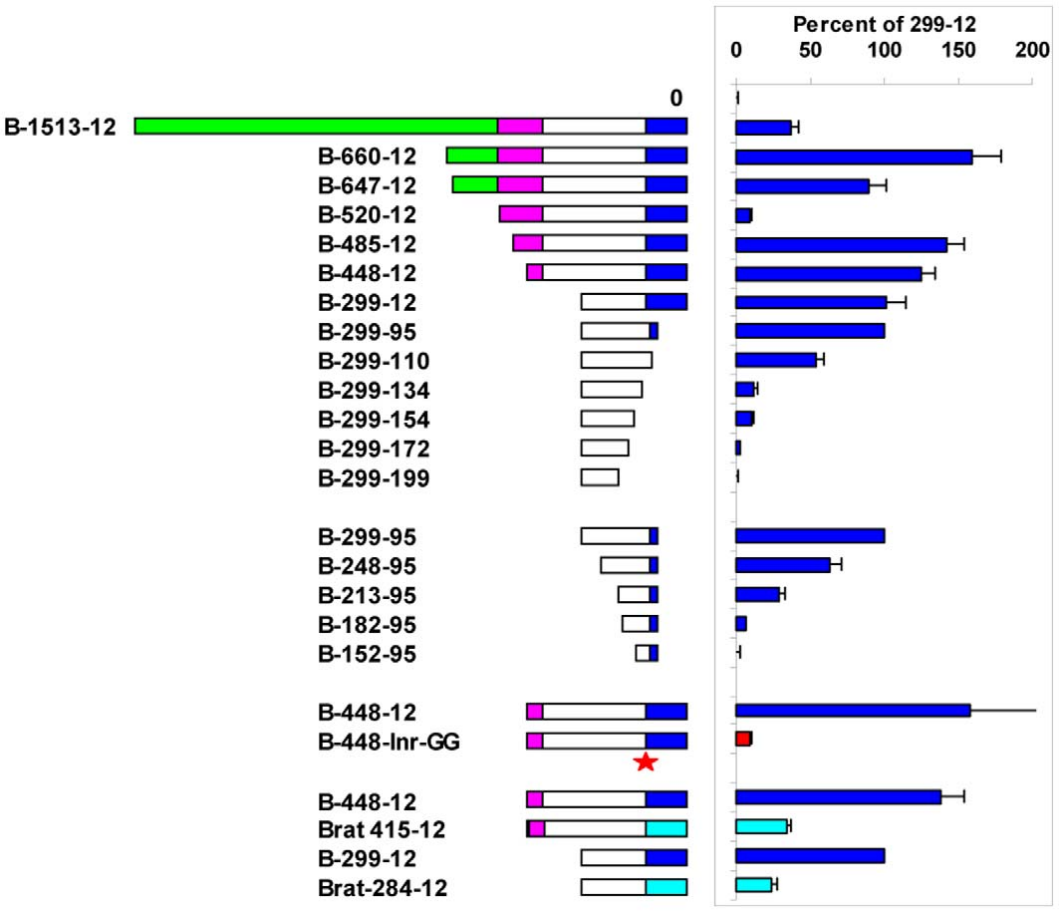
Mapping of promoter activity in the upstream region of the Ly49B gene. pGL4 plasmids containing no insert (0), or various fragments from the upstream region of the *Ly49B* gene were transfected into FSDC cells. Luciferase activity was calculated as a percentage of that observed with the B-299-12 PM fragment. Numbers show the distance of the start and end of each fragment from the exon1/intron1 boundary, and the diagrams provide a graphical representation of the fragments: dark blue, exon 1 (defined as the region downstream of the dominant transcription initiation site); unshaded, region homologous to that found in the same position in other *Ly49* genes; purple, region unique to *Ly49B* (see Figure 1); green, LINE repeat sequence. The B-448-Inr-GG fragment was identical to the B-448-12 fragment but with the two A residues that form the dominant TSSs of the endogenous gene mutated to G residues (shown by a red star). In a separate series of experiments the activity of fragments from the putative rat orthologue of *Ly49B*, *Ly49i8*, (light blue bars) was compared to that of the equivalent FM (B-448-12) and PM (B-299-12) fragments of the mouse *Ly49B* gene. Histograms show the mean values obtained in 3–8 independent experiments with each construct, error bars showing the SEM where this was big enough to display.

The *Ly49B* FM fragment displayed striking activity, 4–6 fold higher than that of the HSV promoter, in the FSDC and DC2.4 cell lines, and moderate activity in the J774 and RAW264 macrophage lines (Figure 6), all of which contain substantial levels of *Ly49B* transcripts. It also displayed moderate activity in the LNK and I2/22 immature NK cell lines (Figure 5), which unexpectedly have been found to express *Ly49B* transcripts [15], and in some cell lines that lacked detectable *Ly49B* transcripts, including P815 and A20 cells (Figure 6), and D^−^ cells (Figure 5). To explore in more detail the promoter activity found in the upstream region of the *Ly49B* gene, a variety of fragments was examined for promoter activity in FSDC cells. Extending the *Ly49B* FM (448-12) fragment upstream by 37 bp (485-12) slightly increased its activity (Figure 7), but extending it another 35 bp upstream (520-12) to just before the start of the large LINE sequence greatly reduced its activity (P = 6.1×10^−6^). This 35 bp sequence, CTTAGTTTAACAGTTAAAAAAAAAGAACTTTAACA, located at the 5′ end of the ∼120 bp upstream sequence that is unique to *Ly49B*, may therefore contain a powerful repressor site. Surprisingly, extending the fragment further upstream, into the LINE region (B-647-12, B-660-12), restored high promoter activity, indicating a complex interplay of regulatory elements in this region of the *Ly49B* gene. Conversely, analysis of truncated PM fragments revealed that core promoter activity resided in an ∼200 bp region (B-299-95) whose 3′ end was just downstream of the major TSS. When this fragment was truncated by a further 15 bp at its 3′ end, so that only the first of the two Inr-like sequences remained (B-299-110), promoter activity was markedly reduced (P = 0.002). Further truncation that removed both Inr-like sequences (B-299-134) reduced activity to low levels (P = 3.9×10^−9^). More importantly, mutation of the two A residues that comprised the actual TSSs within the two Inr-like sequences to G residues (B-448-Inr-GG) essentially eliminated promoter activity. Truncation of the B-299-95 fragment at its 5′ end also reduced activity: removal of the first 51 bp (B-248-95) reduced activity significantly (P = 0.01), and removal of the first 86 bp (B-213-95) reduced activity markedly (P = 7.0×10^−5^). Finally, with a view to eventually identifying the transcription factors that control the expression of *Ly49B*, we examined fragments from the upstream region of the putative rat orthologue of *Ly49B*, *Ly49i8*. However, as shown at the bottom of Figure 7, Brat-415-12 and Brat-284-12 fragments had much lower activity than the corresponding FM and PM fragments of the mouse *Ly49B* gene (P = 0.02 and 0.002 respectively), indicating that the key transcription binding sites in the *Ly49B* gene are not conserved in the rat *Ly49i8* gene.

## Discussion

In recent years the traditional model of vertebrate gene expression in which transcripts originate from fixed positions approximately 30 bases downstream of promoters containing TATA boxes has largely been abandoned in the light of genome wide analyses that have revealed that transcription occurs much more extensively than previously envisaged, that transcriptional units often do not have sharply defined starting points, that genes often contain multiple widely separated TSS clusters, and that upstream regulatory regions frequently lack TATA boxes and/or Inr sequences [38], [39], [40], [41], [42], [43], [44]. In particular, the use of cap-specific cDNA cloning and related CAGE technology to efficiently capture the 5′ ends of transcripts has revealed that although transcriptional initiation of vertebrate genes is sometimes concentrated in a narrow window with a single dominant start site, it is more often spread across a broad window spanning >50 bases [38], [39], [40], [44].

The cap-specific analysis of *Ly49* TSSs described here revealed a striking differential use of these two patterns: *Ly49B* and *Ly49Q* gene expression in myeloid cells, activating *Ly49D* and *Ly49H* gene expression in NK cells, *Ly49F* gene expression in activated T cells, and to a lesser extent *Ly49E* gene expression in vivo, occurs predominantly in a narrow window in the middle of a conserved pyrimidine rich region ∼110 nucleotides upstream of the exon1/intron1 boundary that has the consensus sequence ttctgcCTttcttctt (actual start sites shown in upper case). The *Ly49B* and *Ly49Q* genes also have a second TSS 9 bases further upstream. The finding of, and the location of, dominant narrow TSSs in this subset of Ly49 genes is consistent with several other studies. Firstly, 3 of the 5 TSSs identified in the Riken mouse genome cap-trapper project for this set of genes (shown by green-shaded nucleotides in Figure 1), namely those for Ly49B (AK017140), Ly49Q (AK080021), and Ly49D (AK080171), correspond to the dominant sites found in the present study. Interestingly, the single *Ly49A* sequence found in the Riken project (AK080158) corresponds to the most frequently used *Ly49A* TSS found in the present study, which is only one base downstream from the *Ly49A* TSS in EL4 cells identified by Kubo et al [16] using RNase protection and primer extension. Secondly, in the Riken mouse genome CAGE project [45] only one *Ly49* gene, *Ly49B*, generated a useful number of tags, 33. Of these, 14 had start sites within the above described pyrimidine-rich region, 5 of which corresponded to the downstream TSS identified in the present study, and 4 to the upstream TSS. Finally, a recent RACE analysis of *Ly49H* found the same dominant TSS as in the present study [13].

Although genes using narrow transcriptional windows are more likely to be associated with nearby TATA boxes [40], [44], none of the subset of *Ly49* genes having dominant narrow TSSs contains a site within the first 100 nucleotides upstream that matches the position weighted TATA box matrix in the JASPAR database. By contrast, the two adjacent TSSs in *Ly49B* and the upstream TSS in *Ly49Q* match exactly the canonical Inr consensus sequence YYANWYY [46], and show the highest scores against the JASPAR Inr matrix [47] of any sequence throughout the entire set of sequences displayed in Figure 1A. The Ly49B gene also possesses a precisely positioned consensus DPE promoter element, AGACC [46], 28–32 bp downstream of the dominant TSS. In addition, ∼30 bp upstream of the *Ly49B* TSS is a TA-rich region preceded by CG-rich region that has some resemblance to BRE-TATA elements [46]. However, the significance of these observations is unclear because (a) the corresponding dominant start sites in other *Ly49* genes match neither the Inr consensus sequence nor the JASPAR matrix due to an absence of the central A residue of the motif, (b) the canonical DPE sequence is also present in *Ly49E, H, and Q* but not *Ly49D or F*, (c) multiple relatively high scoring matches to the JASPAR Inr, DPE, and BRE matrices are found throughout the exon 1 sequences of each gene, (d) whole genome analysis shows that even in TATA-less genes many start sites do not conform to Inr sequences [40], (e) the frequency of these motifs in randomized sequences is remarkably high [48].

In striking contrast, transcript initiation in the inhibitory “missing self” *Ly49* genes occurred in several broad transcriptional regions located in at least three widely separated locations, namely upstream of the exon1/intron1 boundary, upstream of exon -1b, and around the intron1/exon2 boundary. These findings are consistent with a previous study which showed a wide dispersal of TSSs in the upstream region of the *Ly49A* gene and around the intron1/exon2 boundary of the *Ly49G* gene [18]. However, our study has extended these findings by revealing that most, and probably all, *Ly49* genes can initiate transcripts in the intron1-exon2 region, and that *Ly49A* initiates a high proportion of transcripts at this location. So too does *Ly49E,* at least in NK cells cultured in vitro in IL2. An unexpected finding was that transcripts could be initiated from downstream of the translational start codon in exon 2, and even from downstream introns and exons. The frequency of this latter event was almost certainly underestimated due to the fact that the reverse RACE primers were not positioned at the 3′ ends of the genes. Even so, at least in the case of *Ly49G* it was clearly relatively common, 13/59 (22%) of *Ly49G* RACE cDNAs having start points downstream of exon 2. Initiation from sites downstream of the start codon in exon 2 was particularly common in *Ly49E*, 68/131 (52%) of transcripts from cultured NK cells being of this type. In *Ly49E* and most other *Ly49* genes the first in-frame ATG downstream of the normal translation initiation codon is in exon 4, so transcripts generated from these sites would yield proteins that could not enter the ER and would presumably be non-functional.

Although the positions of the TSSs in the intron1-exon2 region appeared to be distributed largely at random, there was in fact a remarkable bias favouring A as the start base. Amongst all *Ly49* genes, 145/204 (71%) of transcripts that initiated in this region began with an A, compared with an overall regional frequency of A residues of 31% (P = 1.2×10^−27^). Counteracting this, there was a marked under representation of C and T as start residues (P = 1.9×10^−10^ and 1.7×10^−12^ compared to the regional frequencies). The same highly significant biases persisted even when *Ly49E*, the only *Ly49* to have a dominant TSS in this region, was removed from the analysis, or when start sites were enumerated without any weighting for the number of transcripts at each site. These biases in starting bases are qualitatively similar, but quantitatively much more pronounced, than reported in a genome wide survey in humans [38], and in a genome wide survey in mice which revealed a strong bias for purine start sites [40]. By contrast, the start bases used by transcripts in the exon 1 region did not show significant base biases. Interestingly, none of the *Ly49* genes contains an in-frame ATG triplet downstream of the dominant TSS region in exon 1 nor in the most actively transcribed regions at the 3′ ends of intron 1, nor does *Ly49I* contain an ATG triplet in exon -1b, making it highly likely that the first in-frame ATG triplet in exon 2 is exclusively used as the translation initiation codon regardless of the point upstream at which transcription is initiated.

**Figure 8:**
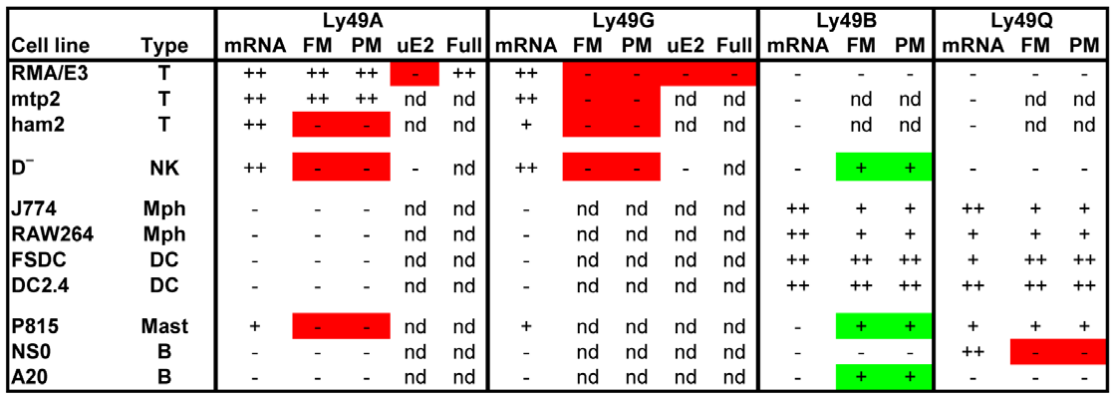
Correlation between the promoter activity of transfected fragments and the expression of endogenous *Ly49* genes. The data summarizes the cell lines of various types (T, T cell; NK, NK cell, Mph, macrophage; DC, dendritic cell; Mast, mast cell; B, B cell) that were examined in this study for the expression of *Ly49* mRNA transcripts and that were transfected with pGL4-luciferase plasmids containing the various upstream fragments (FM, PM, uE2, and Full) described in detail elsewhere in this manuscript. The level of expression is shown as: ++, high; +, clearly detectable; −, marginal or undetectable; nd, not determined. Green boxes highlight situations where promoter activity was detected in cells that lacked expression of the corresponding endogenous gene. Red boxes highlight situations where promoter activity was marginal or undetectable in cells that expressed the endogenous gene.

The key data obtained in this study concerning the promoter activity of *Ly49* gene fragments in various cells and its relationship to Ly49 gene expression in those cells is summarized in Figure 8. Two situations in which there is a lack of concordance between the promoter activity of transfected fragments and the expression of the endogenous gene were identified. Those highlighted in green are where promoter activity occurred in the absence of detectable expression of the endogenous gene. Such situations could occur if the transfected cells expressed some or all of the relevant transcription factors but also expressed repressive factors that bound to sites not present in the transfected gene fragment or if the endogenous gene was in a closed chromatin configuration. Those highlighted in red are where promoter activity was absent in cells that expressed the endogenous gene. Such cells must clearly express all of the required transacting factors, and the absence of promoter activity in such cells demonstrates that the tested fragment by itself does not comprise a functional promoter.

The only *Ly49* gene region tested that showed promoter activity consistent with a role in the expression of the endogenous gene was the upstream region of the *Ly49B* gene, and in particular an ∼200 bp fragment whose 3′ end contained the dominant TSS. This fragment was active in all *Ly49B*-expressing cells, most notably in dendritic cell lines where its activity was many fold greater than the HSV positive control promoter. The region upstream of the *Ly49Q* gene also possessed significant promoter activity in most *Ly49Q*-expressing cells, but not in *Ly49Q*-expressing NS0 cells. By contrast, the corresponding regions from most other *Ly49* genes displayed little or no promoter activity in relevant cells. The main exception to this rule was that fragments from upstream of the *Ly49A* gene often displayed high activity in certain EL4 sublines in agreement with previous reports [17], [19], [21]. However, they lacked activity in the *Ly49A*-expressing ham2 subline of EL4 and, most importantly, upstream fragments from neither the *Ly49A* gene nor other *Ly49* genes displayed any significant promoter activity in the D^−^ NK cell line that expressed multiple members of the *Ly49* family at high levels, including *Ly49A*. Coupled with the absence of a dominant TSS downstream of the putative *Ly49A* promoter and the finding from RACE analysis that transcripts could be initiated internally throughout the length of the putative core (PM) *Ly49A* promoter fragment, it appears likely that the high promoter activity of *Ly49A* upstream fragments in some EL4 sublines is caused by spurious transcription factor expression in EL4 sublines that is unrelated to the expression of the endogenous gene or to the physiological control of *Ly49* gene expression in NK cells. Similarly, no significant promoter activity could be detected in fragments of various sizes surrounding the commonly used transcription initiation region in intron1/exon 2. Taken together, there appears to be no evidence for the existence of classical promoters with significant tissue specific activity in the so-called Pro2 and Pro3 regions [23] of the *Ly49* genes expressed in lymphoid cells.

Instead, our studies showed a striking and consistent difference in the pattern of transcriptional initiation between those *Ly49* genes that are expressed in a stochastic manner (A, C, E, G, and I) and those that are probably not (B, D, H, and Q). Intriguingly, the former genes all possess a conserved upstream Pro1 region, whereas the latter genes either lack a recognizable Pro1 region (*Ly49B* and *Ly49Q*) or have a substantially different and apparently non-functional Pro1 sequence (*Ly49D* and *Ly49H*) ([23], [24], and unpublished data). Direct evidence for the importance of this region is provided by the finding that it forms a DNase I hypersensitivity site in vivo, and its deletion altered the pattern of expression of an *Ly49* genomic transgene [49]. It has been suggested that Pro1 acts as a bidirectional promoter in immature NK cells, and that when transcription is initiated in the forward direction it neutralizes a repressor site upstream of Pro2 that allows transcription to transfer to Pro2 in mature NK cells [24]. Our finding that transcription in Pro1-containing genes is initiated from multiple widely dispersed regions that lack defined promoter or repressor sites suggests that this model is not correct. Instead we suggest that Pro1 acts by increasing the probability of stable transcription being established from multiple favoured regions throughout the gene, some of which, as strikingly seen in the case of *Ly49E*, may reside downstream of the translational start codon. According to this model, the Pro1 element acts as a developmentally programmed enhancer. Stochastic Ly49 gene expression occurs if this element is able to stabilize transcription from an appropriate downstream site within a developmentally programmed time window, by physically interacting with that site and/or facilitating the binding of transcription factors there. Failure to stabilize any site within the gene or the selection of an inappropriate site downstream of the translational start codon results in a non-productive allele in mature NK cells. The distinctive Pro1 region found in the genes encoding activating Ly49s such as Ly49D and H may preferentially stabilize transcription from the conserved default initiator region in exon 1 thereby reducing the degree of stochastic expression [12] and increasing the probability of biallelic expression [13]. Transcription from Pro1 itself may be needed to open up the gene to transcription factors, or may simply indicate that it is another favoured initiation region.

## Supporting Information

Figure S1 **Location of reverse primers used for RACE PCRs.** Exons are shown in alternate blue and green.
(PDF)

Table S1 **Sequences of primers.**
(PDF)

Table S2 **Sequences of promoter fragments.** EcoRV and HindIII cloning sites are shown in lower case and underlined. Exonic sequences are highlighted in blue, LINE sequences in green, the unique sequence found in Ly49B in grey, and translation start codons and mutations in red.
(PDF)
