# Supplementary figures and images for: Comprehensive Analysis of Transcript Start Sites in Ly49 Genes Reveals an Unexpected Relationship with Gene Function and a Lack Of Upstream Promoters
Source: PLoS One. 2011 Mar 31;6(3):e18475. doi: 10.1371/journal.pone.0018475 (PMC3069108; doi:10.1371/journal.pone.0018475)

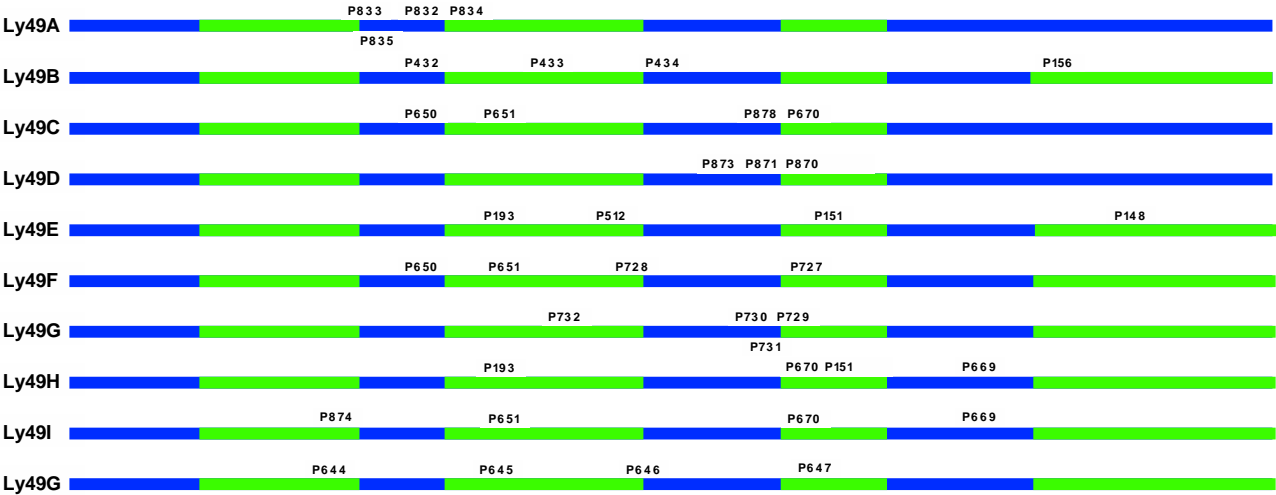

Supplement: Figure S1 — Location of reverse primers used for RACE PCRs. Exons are shown in alternate blue and green. (PDF) [file pone.0018475.s001.pdf]
