# Supplementary material for: Comprehensive Analysis of Transcript Start Sites in Ly49 Genes Reveals an Unexpected Relationship with Gene Function and a Lack Of Upstream Promoters
Source: PLoS One. 2011 Mar 31;6(3):e18475. doi: 10.1371/journal.pone.0018475 (PMC3069108; doi:10.1371/journal.pone.0018475)
Supplement: Table S1 — Sequences of primers. (PDF) [file pone.0018475.s002.pdf]

Supplementary Table 1. Sequences of primers

		**RACE reverse primers**
Ly49A	P832	CACTGAAACAGCTACCAGAA
	P833	CCAGTGGAATGAACACCTT
	P834	GGTTTAGAAATTCCTGCAGTTT
	P835	CAATGAACTTCCAGTGGAAT
Ly49B	P156	TAACTTTCATCTTCATCCCTCTGC
	P432	CACCAACACTGCCACAGTT
	P433	GGCCTTACACTCTGAAGA
	P434	CAGCAGAACCAGTATCCT
Ly49C	P650	ACTGCCAACACTGCAACT
	P651	GCATGTTGCTGCAGTTATG
	P670	GGAATAACATGGCGTTGAA
	P878	CTTCAGAATGGGAACGCTAT
Ly49D	P870	GAAGGAACCACGAGCTGAA
	P871	CTTCAGTTCATCCTCATCAT
	P873	GAAGGGTTAAGCTGGAATT
Ly49E	P148	TCTGTCTCCAAGAGGAAGG
	P151	CATATGACAATCCAATCCAG
	P193	TCACTTTGCATGTTGCTGCAG
	P512	CACTGTACCATCTGTTCTGTTC
Ly49F	P650	ACTGCCAACACTGCAACT
	P651	GCATGTTGCTGCAGTTATG
	P727	GTCTGGAATAACCTGGAAT
	P728	CCTGTGAGGAATCTAAATCT
Ly49G	P729	GTCTGAAGGAGCCAGGTT
	P730	GAAGGGATAAGCTGGAAAT
	P731	CTTCAGTTCATCCTCATTGT
	P732	CATTGCCTGGCCTACACT
Ly49H	P151	CATATGACAATCCAATCCAG
	P193	TCACTTTGCATGTTGCTGCAG
	P669	CCACAAATACAGTAGTAGGGAATA
	P670	GGAATAACATGGCGTTGAA
Ly49I	P651	GCATGTTGCTGCAGTTATG
	P669	CCACAAATACAGTAGTAGGGAATA
	P670	GGAATAACATGGCGTTGAA
	P874	CTTTATCGTCAACACTGCAACTA
Ly49Q	P644	CCTCAGGCCTCACTTGGTT
	P645	GCATGGTGCTACAGTTAT
	P646	GGCTTCCACCTGGGTGCTT
	P647	CCAGTAACTGTCTGGAGTA

RT-PCR primers

Ly49A-E1	P886	CCACTTCTTGCTAGCGACA
Ly49A-E2	P890	ATGAGTGAGCAGGAGGTCA
Ly49A-rev	P872	GAAGGGATAAACTGGAACT
Ly49B-E1	P431	CCCTTCACCAGAATTACTT
Ly49B-rev	P432	CACCAACACTGCCACAGTT
Ly49E-E4	P98	CATGGATCCGCCACAACCATAACTGCAGC
Ly49E-rev	P148	TCTGTCTCCAAGAGGAAGG
Ly49G-E1	P886	CCACTTCTTGCTAGCGACA
Ly49G-E2	P890	ATGAGTGAGCAGGAGGTCA
Ly49G-rev	P729	GTCTGAAGGAGCCAGGTT
Ly49Q-E1	P160	CTTTTCCCCCATAACTGCAG
Ly49Q-rev	P161	TGACCTCTTCCAGCTCTTCC
Actin-fwd	P507	ATCCTGACCCTGAAGTACCC
Actin-rev	P508	TACTCCTGCTTGCTGATCC
