# Supplementary material for: Comprehensive Analysis of Transcript Start Sites in Ly49 Genes Reveals an Unexpected Relationship with Gene Function and a Lack Of Upstream Promoters
Source: PLoS One. 2011 Mar 31;6(3):e18475. doi: 10.1371/journal.pone.0018475 (PMC3069108; doi:10.1371/journal.pone.0018475)
Supplement: Table S2 — Sequences of promoter fragments. EcoRV and HindIII cloning sites are shown in lower case and underlined. Exonic sequences are highlighted in blue, LINE sequences in green, the unique sequence found in Ly49B in grey, and translation start codons and mutations in red. (PDF) [file pone.0018475.s003.pdf]

Supplementary Table 2. Sequences of promoter fragments

HSV

gatatcGGATCAAGATCCTGCTTCATCCCCGTGGCCCGTTGCTCGCGTTTGCTGGCGGTGTCCCCGGAAGAAATATATTT
GCATGTCTTTAGTTCTATGATGACACAAACCCCGCCCAGCGTCTTGTCATTGGCGAATTCGAACACGCAGATGCAGTCGG
GGCGGCGCGGTCCCAGGTCCACTTCGCATATTAAGGTGACGCGTGTGGCCTCGAACACCGAGCGACCCTGCAGCGACCCG
CTTAACAGCGTCAACAGCGTGCCGCAGATCaagctt

FM fragments

A-FM [430-12]
gatatcGCTGCAACTAGCATAATTCAAAATGAAGAAATAACTTAAAAATTAGTAACATAATAGTAAATTTCCAACAGTCT
ATTACAAAACTAGGAGCATCAGTGCCACATTTTTCCAAGTTGTTTTGCTAAGGACAGTGTTTTTGCTCTCTCTGCAGGAG
TTTTCATCTTCTCCTTCTTAAAAATATCAGTTATGGACATTTGTTTTGCAGCATCTGGCACAATACGTTACTTCTCTCCT
TTGTTCTGAGGGTCAGGTTTCATTAAGCAGTTTCCTCTTTTTGCTTTGATGACGAGGAGGAGCATAAAATCATGAGGTTG
AGTATCTCTCAGTGGAAATTTAGTTCTACCGTTTATTTTGGAGACACTTAGGGGATATCAACCAGAAAAAGCCAACTTTT
TCCTCCACCAGAACCACTTCTTGCaagctt

B-FM [448-12]
gatatcGGTACAATAAGTACAATTCAACAACAAGTAAACATTTTCAAATAGAAAAAGAAAATTTCCAACAGCCTATTACA
AAATTAGAAGCAGGGGAATTTCAGTCCCCCATTCTTTTCCTATGGTTTTGTTAAGGACACTTATTTTTCTTTCTCTGAAG
GAGTGGTATCTTTTCAGTATTCAACTATCAATTATGGAAATCTATTGCAGTATATGCCAGAATACATTGGTCCTTTCCTT
TGCTGTCAGAGTCAGCTTTTATTATGCAATTTCCTCTTTTTGCTTAGATCAAGAGGAGGGGCCGAATGTTATGAGCTGGA
GTATTACTCAGAGATGATTCAGTTCCGTCATTCTTCTTGAAGCTCTGAGTGTATACAGACCAGGAATGGCCAACTTGTCC
CTCAGTTAGTGGATTAATTCCCTTCACCAGAATTACTTCCTGGaagctt

C-FM [420-12]
gatatcGCTGCAATTAGTATAATTCAAAATCAAGGAGTAACTAAAAAAATAGTAACATAAGAGTAAATTTCAAACAGTCT
ATGACAAAATTAGCCTACGAACTTCAGTCCCTCTTTTTCCTATTTGTTTTGCTAAAGACAATGTTTTCCCCTCTCTGCAG
CAGTGTGCATCTTCTCTATCTTCAAATATAAATCATTCACATTTGTTTTGTCCATCCAATACTATATGTTGTTTCAGATT
GCAATAAGCAATTTCCTCTTTTTGCTTTGGTGACGAGGAGGGGCAGAAAATCATGAGGTTGAGTATCACCCGGTGGAAAT
TTAGTTCCGACTTTCAATTTTGAAACTCGTAGGAGATCTAAACCAGAAAACGCCAACGTTTCAGACAAATTTTCCCTCCA
CCAGCATCACTCCGGaagctt

D-FM [455-12]
gatatcGATGCAATTAGCATGATTCAACATCAAAACATTAATATAAAAAATAGCAACATAATAGAAGATTTCCAAAAGTC
GAATAAAAAATTGTGAGCAGACAAGTTTTAAGTCCATCTTTTTCCCAATGGTTTTGCTAAAGACACAGGTTTTGCTCTCT
GTGCAGGAATGCTCATCTTTTCTGTCTTCAAATATCATATATAGTCATTTCTTTTGCAGCATCTGGCAAAATATTTTGCT
TCTTTCCCTTGCCTTCAGACTCAGCTTTCAAAAGCAATTTCCTCTTTTTGATTTGGTCAAGAGGAGGGGCAGAAAACCAT
GAGATTGAGTGTTGCTCAGAGGAAATTTAGTTCTGCCTTTCTTCTTGGAGCCTCTAAGGGGATACACACCAGAAAAGGCC
CACATTACCCCAACAGGGACATCCATTCCTTCTACCCACCTCACTTCAGGaagctt

E-FM [421-12]
gatatcGCTGCAATTAGCATAATTCAAAATCAAGGAGTAACTAAAAAATATAGTAACATAAGAATAAATTTCAAACAGTC
TATGACAAAATTAGCCTACAAACTTCAGTCCCTCTTTTTCCTATTTGTTTTGCTAAAGGCAGTGTTTTGCCTTCTCTGTA
GGAGTGTCTATCTTCTCTGTCTTAAAATATAAATTATTCACATTTGTTTTGTCCACCTGATACTATATGTTGTTTCAAAT
TGCAATAAGCAATTTCCTCCTTTTGCTTAGATAAAGAGGAGGGGCAGAAAATCATGAGGTTGAGTATCACTAGGTGGAAA
TTTAGTTCCGTCTTTCATTTTTGAAACTCGTAGGGGATATAGACCAGAAAACGCCAACTTTTCACCCAACTTTTCCCTCC
ACCAGAATCACTCCGGaagctt

F-FM [418-12]
gatatcGCTGCAATTAGCATAATTCTAATTCAAGGAATAACTAAAAAAATAGTAACTTAAATGTAATTTCCAACAGTCTA
TGACAAAATTAGCCTACGAACTTCTCTCCCACTTTTTCCTATTTGTTTTGCTAAAGACAATGCTTTCTCCTCTCAGCAGG
AGTGTCCATCATCTCTGTCTTCAAATATAAATTATTCACATTTGTTTTGTCCACCTGATACTATATGTTGTTTCAGATTG
CAATAAGCAATTTCCTCTTTTTGCTTTGATGAAGAGGAGGGGCAGAAAATCATGAGGTTGAGTATCACTCGGTGGAAATT
TAGTTCTGTCTTTCATTTTTGAAACTTGTAGGGGATATAGACTAGAAAACACCAACTCTACAGACAAATTTTCCCTCACC
AGAATCACTCCGGaagctt

G-FM [462-12]
gatatcGCTGCAATTAGAATAATTCAAAATGAAGAAATAACTTAAAAATTAGAAACATAATAGTAAATTTCCAAAATTAC
AAATTACAAATTATAATTTAAATTACAAATTATAATTACAAAACAAGGAGCATCAGTCCCACATTTTTCCTAGTTGTTTT
GCTAAGGATAGTGTTTTTGCTCTCTCTGCAGGAGTGTTCATCTTCTCTGTCTTAAAAATATCAGTTATGGACATTTGTTT
TGCAGCATCTGGCACAATATGTTTCTTCTCTCCTTTGTTCTGAGGGTCAGGTTTCATTAAGCAGTTTCCTCTTTTTGCTT
TGATGAAGAGGAGGAGCATAAAATCATGAGGTTGAGTATCACTCAGTGGAAATTTAGTTCTACTGTTTATTTTGGAGACA
CTTAGGGGATATCAACCAGAAAAAGCCAACTTTTTCTCCACAGGAATCACTTCTCAGaagctt

H-FM [385-12]
gatatcTATTTTTAAAAAAATTTGGAGCACAGAATCTTCAGTCCCTCTTTTTCATATTTGATTTGCTGAGGACACATGT
TTTGCTCTCTCTGGTGGAGTGCTCATCTTCTCTGTCTTCAAATATCATTTATAGATGTTTCTTTTGCAACATCTGGCCGA
ATATTTTGCTCCTTTAATTTGTTCTCAGACTCAGATTTCAAAAGCAATTTCCTCTTTTCGATTTGGTCAAGAGGAGGGGC
AGAAAAACATGAGGTTGAGTATCACTCGGAGGAAATTTAGTTCTGCCTTTCTTCTTGGAGCCTCTTAGGGGATACAGACC
AGTAAAGGCCCACATTACCCCAATTGAGGCATCCATTCTTTCTACCGGCATCACTTCAGGGaagctt

I-FM
gatatcGCTGCAATTAGCATAATTCAAAATCAAGGAGTAACTAAAAAATAGTAACATAAGAGTAAATTTCAAACAGTTTA
TGACAAAATTAGCCTATGAACTTCAGTACCTCTTTTTCCTATTTGTTTTGGTAAAGACAATGTTTTCCCCTCTCTGCAGG
AGTGTGTCTTCTCTATCTTCAAATATAAATTATTCACATTTGTTTTGTCCATCCAATACTATATGTTGTTTCAGATTGCA
ATAAGCAATTTCCTCTTTTTGCTTTGTTGACAAGGAGGGGCAGAAAATCACGAGGTTGAGTATCACTCGGTGGAAATTTA
GCTCTGTCTTTCAATTTTGAAACTCATAGGAGATATAGACCAGAAAAACGCCAACTTTTCAGACAAATTTTCCCTCCACC
AGAATCACTCCGGaagctt

Q-FM [459-12]
gatatcGCTGCAATTAGCATAACTCAAAATCAAAGAGTAACTTAGAAAACAGTAACATAAGAGAAATTTTTCAACAGCCT
ATTACAAACCTGGAGCTGAGGAACCTGAGCTGCACATTTTTCTAGTTGTTTTGCTAAGGACACTGGTTTTGCTCTCTCTG
CAGCAGTGTTCATCTTCTCAGTCTTCAAGTTTCAATTATGGACATTTCTTTTGCAGCATCTGGCACAATATGTTACTTCT
TTCCTTTGCTTTCAGAGTCAGGTTTCATTAAGCAATTTCCTCTTTTCGATTTGGTCAAGGAGGAGGGGCAGAAAATCATG
AGATAGAGTATCACTCAGAGGAAAAAATTAGTTCTGCCTTTCTTCTTGGAGCCTCTTACAGGATAGAGACCAGGAAAGAC
AACTTTTCCCCCATAACTGCAGGATCTATTATCTTCACCAGAATCTCTTCAGGGaagctt

PM fragments

A-PM [283-12]
gatatcTGCAGGAGTTTTCATCTTCTCCTTCTTAAAAATATCAGTTATGGACATTTGTTTTGCAGCATCTGGCACAATAC
GTTACTTCTCTCCTTTGTTCTGAGGGTCAGGTTTCATTAAGCAGTTTCCTCTTTTTGCTTTGATGACGAGGAGGAGCATA
AAATCATGAGGTTGAGTATCTCTCAGTGGAAATTTAGTTCTACCGTTTATTTTGGAGACACTTAGGGGATATCAACCAGA
AAAAGCCAACTTTTTCCTCCACCAGAACCACTTCTTGCaagctt

B-PM [299-12]
gatatcTGAAGGAGTGGTATCTTTTCAGTATTCAACTATCAATTATGGAAATCTATTGCAGTATATGCCAGAATACATTG
GTCCTTTCCTTTGCTGTCAGAGTCAGCTTTTATTATGCAATTTCCTCTTTTTGCTTAGATCAAGAGGAGGGGCCGAATGT
TATGAGCTGGAGTATTACTCAGAGATGATTCAGTTCCGTCATTCTTCTTGAAGCTCTGAGTGTATACAGACCAGGAATGG
CCAACTTGTCCCTCAGTTAGTGGATTAATTCCCTTCACCAGAATTACTTCCTGGaagctt

C-PM [271-12]
gatatcTGCAGCAGTGTGCATCTTCTCTATCTTCAAATATAAATCATTCACATTTGTTTTGTCCATCCAATACTATATGT
TGTTTCAGATTGCAATAAGCAATTTCCTCTTTTTGCTTTGGTGACGAGGAGGGGCAGAAAATCATGAGGTTGAGTATCAC
CCGGTGGAAATTTAGTTCCGACTTTCAATTTTGAAACTCGTAGGAGATCTAAACCAGAAAACGCCAACGTTTCAGACAAA
TTTTCCCTCCACCAGCATCACTCCGGaagctt

D-PM [301-12]
gatatcGTGCAGGAATGCTCATCTTTTCTGTCTTCAAATATCATATATAGTCATTTCTTTTGCAGCATCTGGCAAAATAT
TTTGCTTCTTTCCCTTGCCTTCAGACTCAGCTTTCAAAAGCAATTTCCTCTTTTTGATTTGGTCAAGAGGAGGGGCAGAA
AACCATGAGATTGAGTGTTGCTCAGAGGAAATTTAGTTCTGCCTTTCTTCTTGGAGCCTCTAAGGGGATACACACCAGAA
AAGGCCCACATTACCCCAACAGGGACATCCATTCCTTCTACCCACCTCACTTCAGGaagctt

E-PM [271-12]
gatatcTGTAGGAGTGTCTATCTTCTCTGTCTTAAAATATAAATTATTCACATTTGTTTTGTCCACCTGATACTATATGT
TGTTTCAAATTGCAATAAGCAATTTCCTCCTTTTGCTTAGATAAAGAGGAGGGGCAGAAAATCATGAGGTTGAGTATCAC
TAGGTGGAAATTTAGTTCCGTCTTTCATTTTTGAAACTCGTAGGGGATATAGACCAGAAAACGCCAACTTTTCACCCAAC
TTTTCCCTCCACCAGAATCACTCCGGaagctt

F-PM [270-12]
gatatcAGCAGGAGTGTCCATCATCTCTGTCTTCAAATATAAATTATTCACATTTGTTTTGTCCACCTGATACTATATGT
TGTTTCAGATTGCAATAAGCAATTTCCTCTTTTTGCTTTGATGAAGAGGAGGGGCAGAAAATCATGAGGTTGAGTATCAC
TCGGTGGAAATTTAGTTCTGTCTTTCATTTTTGAAACTTGTAGGGGATATAGACTAGAAAACACCAACTCTACAGACAAA
TTTTCCCTCACCAGAATCACTCCGGaagctt

G-PM [283-12]
gatatcTGCAGGAGTGTTCATCTTCTCTGTCTTAAAAATATCAGTTATGGACATTTGTTTTGCAGCATCTGGCACAATAT
GTTTCTTCTCTCCTTTGTTCTGAGGGTCAGGTTTCATTAAGCAGTTTCCTCTTTTTGCTTTGATGAAGAGGAGGAGCATA
AAATCATGAGGTTGAGTATCACTCAGTGGAAATTTAGTTCTACTGTTTATTTTGGAGACACTTAGGGGATATCAACCAGA
AAAAGCCAACTTTTTCTCCACAGGAATCACTTCTCAGaagctt

H-PM [301-12]
gatatcTGGTGGAGTGCTCATCTTCTCTGTCTTCAAATATCATTTATAGATGTTTCTTTTGCAACATCTGGCCGAATATT
TTGCTCCTTTAATTTGTTCTCAGACTCAGATTTCAAAAGCAATTTCCTCTTTTCGATTTGGTCAAGAGGAGGGGCAGAAA
AACATGAGGTTGAGTATCACTCGGAGGAAATTTAGTTCTGCCTTTCTTCTTGGAGCCTCTTAGGGGATACAGACCAGTAA
AGGCCCACATTACCCCAATTGAGGCATCCATTCTTTCTACCGGCATCACTTCAGGGaagctt

I-PM [270-12]
gatatcTGCAGGAGTGTGTCTTCTCTATCTTCAAATATAAATTATTCACATTTGTTTTGTCCATCCAATACTATATGTTG
TTTCAGATTGCAATAAGCAATTTCCTCTTTTTGCTTTGTTGACAAGGAGGGGCAGAAAATCACGAGGTTGAGTATCACTC
GGTGGAAATTTAGCTCTGTCTTTCAATTTTGAAACTCATAGGAGATATAGACCAGAAAAACGCCAACTTTTCAGACAAAT
TTTCCCTCCACCAGAATCACTCCGGaagctt

Q-PM [307-12]
gatatcTGCAGCAGTGTTCATCTTCTCAGTCTTCAAGTTTCAATTATGGACATTTCTTTTGCAGCATCTGGCACAATATG
TTACTTCTTTCCTTTGCTTTCAGAGTCAGGTTTCATTAAGCAATTTCCTCTTTTCGATTTGGTCAAGGAGGAGGGGCAGA
AAATCATGAGATAGAGTATCACTCAGAGGAAAAAATTAGTTCTGCCTTTCTTCTTGGAGCCTCTTACAGGATAGAGACCA
GGAAAGACAACTTTTCCCCCATAACTGCAGGATCTATTATCTTCACCAGAATCTCTTCAGGGaagctt

uE2 fragments

G-824-1
gatatcCATGAACATGAAATGCTGTGAGATTAAAAGTTCATAATGTATCCTCTATGCCTCTCTCTGTTCCTCCCACACCC
CATTCAGCAATGACCCCTTTCTTCTTCTCTGTTTGCTGTCGGTACTAAAGGATAGACATCATTTCTGCTGACTTTGAGCT
ATGGGACTCAGATGATATAGAATATACCACATTTTTCTTTCTGGGTCTGGTTTATCCCACTCATTAGGAACTTTTCTTGT
TGAATCTACTTACCCGGTTGAAGCATGGAGTAGAGTCTAGAGAATGTGGCTGGGTGGAGAGGTTAGACATTAGCTCATTG
GGGCTTCAATCCAAGAGATATTTATGTATATTGGATCACCTGTAGTATTAACCTTCAAATACCTTTCTTAAGGTAGTGTC
AGGGAAACTCTGACAGTCATGTATAAATAAAAAGACGGAAAAACAATTAATATTCATTTATTATCAAAATGCTTAAATAA
TTTTAAAGCAACATTTTTATTTATTCCTAAAATTTCAAAGAGATTATATCAATTGTAGAATGACTTATTAAATTTTTAAT
TCTTTCCATATTTCTTAGAAATAATTGCTGTGGCCATTATAAACGACTTTAACAAAAATCTACAATGAGTTCTCACCTGT
AACGGAAATTTGGTCAGTCCATGTCAGGGTGTTTATAGCATTAAGTGAGTTAGTCAGACCCCACCCTTTCCCAGACCTCT
GTATCATCATATCTAGTTATCTTCCTATAGGTGAACATTTTAACATTTTTCGTAGAAACCACTCAAGGCACCATTTTAAC
TGAGAACATACTTCATACATCATTCCCAAGaagctt

G-575-1
gatatcGGTTGAAGCATGGAGTAGAGTCTAGAGAATGTGGCTGGGTGGAGAGGTTAGACATTAGCTCATTGGGGCTTCAA
TCCAAGAGATATTTATGTATATTGGATCACCTGTAGTATTAACCTTCAAATACCTTTCTTAAGGTAGTGTCAGGGAAACT
CTGACAGTCATGTATAAATAAAAAGACGGAAAAACAATTAATATTCATTTATTATCAAAATGCTTAAATAATTTTAAAGC
AACATTTTTATTTATTCCTAAAATTTCAAAGAGATTATATCAATTGTAGAATGACTTATTAAATTTTTAATTCTTTCCAT
ATTTCTTAGAAATAATTGCTGTGGCCATTATAAACGACTTTAACAAAAATCTACAATGAGTTCTCACCTGTAACGGAAAT
TTGGTCAGTCCATGTCAGGGTGTTTATAGCATTAAGTGAGTTAGTCAGACCCCACCCTTTCCCAGACCTCTGTATCATCA
TATCTAGTTATCTTCCTATAGGTGAACATTTTAACATTTTTCGTAGAAACCACTCAAGGCACCATTTTAACTGAGAACAT
ACTTCATACATCAtTCCCAAGaagctt

A-923-1
gatatcCATGAACATGAAATGCTGTGAGATTAAAAGTTCATAATGTATCCTCTAAGACTCTCCCTGTTGCTCTCACACCC
CATCCAGCAATGATCCCTTTCTTCTTCTCAGTTTGCTGTCGGTACTAAAGGATAGAAATCATTTCTGCTGACTTTGAGCT
GTGAGAATCAAATGATATAGAATATATCACATTTTTTTTTCTGGGTATGGTTTAACCCACTCATTGGGAACTTTGCTGGTT
GAATCCACTTACCTGGTTGAAGCATGGAGATGAATGTATAGAAAGTGGCTGGGTGGAAAGTTTGGATATCTTAGCTGATT
AGGGCTTCAGTACAACAAATATTTACGTATGTTGGATCACCTTCAGTATTAACCTTCAAATACCTTTCGTAAGGTACTGT
CAGGGAAACTCTGACAGTCATGTATAAATAAAAAGACAGAAAGAACAATTACTATTCATTTATTATCAAAATGTATAAAT
AATTTTAAAGCAACATTTTTATTTACTCCTAAAATTTCAAAGAGGCTATAACAATTATAGAATGACTTAGGAAATTTTTA
ATCTTTCCATATTTCTTAGAAATAAGTGGGTGGGTATGGGGGACTTTTGGTATAGCATTGGAAATGTAAATGAGCTGAGC
TAAATACCTAATAAAAAATGGGAAAAAAAAGAAATAAGTGCTGTGACCATTAGAAATATAAACTACTTTAATAAAAATCT
ACAATGAGCTCTCACTTGTAATGTAAATTGGGTCAGTCCATGTCAGTGTGTTTGTAGCATTAAGTGAGTTAGTCAGACCC
CACCTTTTTCCAGCCCTCTGTATCATCAGATTCAGTTCTCTTCCTATAGGTGAACATTTTAATATTTTTCTTAGAAACCA
CTCGAGGCACCATTTGAACTGAGAACATACTTTATATATCAATCCCAAGaagctt

A-675-1
gatatcGGTTGAAGCATGGAGATGAATGTATAGAAAGTGGCTGGGTGGAAAGTTTGGATATCTTAGCTGATTAGGGCTTC
AGTACAACAAATATTTACGTATGTTGGATCACCTTCAGTATTAACCTTCAAATACCTTTCGTAAGGTACTGTCAGGGAAA
CTCTGACAGTCATGTATAAATAAAAAGACAGAAAGAACAATTACTATTCATTTATTATCAAAATGTATAAATAATTTTAA
AGCAACATTTTTATTTACTCCTAAAATTTCAAAGAGGCTATAACAATTATAGAATGACTTAGGAAATTTTTAATCTTTCC
ATATTTCTTAGAAATAAGTGGGTGGGTATGGGGGACTTTTGGTATAGCATTGGAAATGTAAATGAGCTGAGCTAAATACC
TAATAAAAAATGGGAAAAAAAAGAAATAAGTGCTGTGACCATTAGAAATATAAACTACTTTAATAAAAATCTACAATGAG
CTCTCACTTGTAATGTAAATTGGGTCAGTCCATGTCAGTGTGTTTGTAGCATTAAGTGAGTTAGTCAGACCCCACCTTTT
TCCAGCCCTCTGTATCATCAGATTCAGTTCTCTTCCTATAGGTGAACATTTTAATATTTTTCTTAGAAACCACTCGAGGC
ACCATTTGAACTGAGAACATACTTTATATATCAATCCCAAGaagctt

A-372-1

gatatcTTAATCTTTCCATATTTCTTAGAAATAAGTGGGTGGGTATGGGGGACTTTTGGTATAGCATTGGAAATGTAAAT
GAGCTGAGCTAAATACCTAATAAAAAATGGGAAAAAAAAGAAATAAGTGCTGTGACCATTAGAAATATAAACTACTTTAA
TAAAAATCTACAATGAGCTCTCACTTGTAATGTAAATTGGGTCAGTCCATGTCAGTGTGTTTGTAGCATTAAGTGAGTTA
GTCAGACCCCACCTTTTTCCAGCCCTCTGTATCATCAGATTCAGTTCTCTTCCTATAGGTGAACATTTTAATATTTTTCT
TAGAAACCACTCGAGGCACCATTTGAACTGAGAACATACTTTATATATCAATCCCAAGaagctt

B-329-1

gatatcAATGATTTATGGTGGTAGCAATTTTGCCTAACTTTTTCATGTATGTAGAATATTTTGAACAAAGGCAGTTTGAA
AATAGTAATGACTTATTCTTATTTTTAAAGTTCTTTCCATTTAAGCAGATTATATGCAAAGAGGTCTCACTTGTGTGTGA
GGAAATCAGAGTCAGGGTGTTTGGACCACTAAGAAAATTAATCATGTCTCACCCATTCCCACTCCAATGCAGCATCAGAT
CCAGGTATCTTCCTAAAGATGGACATTTTAACTTTTTCTTTAGAAATCACTTGAGGCCACATTTTAATACAAATCGTACT
TTACATACTTCCAAGaagctt

C-359-1

gatatcAATGATTTTTTTAGTGTTTTTATGTGCTGAATATTTTGAGCAAACAGACAGTATCAATGGTAGAGTGATTTAGT
AAAGTTTTAATCTTTTTCATATTTCTTAGAAATAATTGCTATTACCATTAGAAAGATAAACTAACTTAACAGAAATCTAC
AATGAGTTCTTATCTGTGATGGAAATTGGGATCAGTGCATGTCAGGGTGTTTGGATTATTAAGTGAGTTAGTCAGACTCT
ACCCTTTTTAATTTCTCTGTATCATCATATCCAGTTATGTTCCCATAGGTGAACATTTTAACATTTTTCTTAGAATTCAT
TGAAATCCCCATTTTACCAACGAACAGACTTCTTGtACTCCCACGaagctt

E-376-1

gatatcAATCATTTTTAGTGGTACCAATTTTACCTAATATTTCTCTTGCTAAATATTTTGAACAAATAGGCTGTATCAAT
GGTAGAATGGCATAGTAAATTTTTAATTTTTTTTCATATTTCTTAGGAATAATTGCTGTTACCATTAGAAATTTAAACAA
CTTTAGCAGAAATCTACAATGAGTTCTCACATGTAATGGAAATCGGGATCAATCTATGTCAGGGCGTTTGGATTATTAAG
TGAGCTAGTCAGACTCTACATTTTTCCAACCCTCTGTCTCATCAGCTTCAGTTATCTTCCTATAGGTGAACACTTTAACA
TTTTTCTTAGAATTCACTCAAAGGCCCATTTTACCACAGAACATACTTCATGCACTCCCACGaagctt

J-350-1

gatatcAATGATTTTTTTAGTGTTTTTCTGTGCTGAATAGTTTGAAAAAATAGGCTTTATCAATGGTAGAGTGATTTAGT
AAAGTTTTAATCTTTTTCATATTTTTTAGCAATAATTGCTATTACCATTAGAAATATAAACTTAACAGAAATCTACAGTC
TTACCTGTGATGGAAATTGGGATCAGTCCATGTCAGGGTATTTGGATTATTAAGTGAGTTAGTCAGACTCTACCCTTTTT
AATCTCTCTGTATCATCATATCCAGTTATGTTCCCATAGGTGAACATTTTATCATTTTTCTTAAAATTCACTGAAATCTC
CATTTTACCAGAGAACAGACTTCTTGTACTCCCACGaagctt

Full length Exon1 - Exon2 fragments

[Exon 1 and exon 2 highlighted in blue, translational start codon in red]

A-429-39

gatatcGCTGCAACTAGCATAATTCAAAATGAAGAAATAACTTAAAAATTAGTAACATAATAGTAAATTTCCAACAGTCT
ATTACAAAACTAGGAGCATCAGTGCCACATTTTTCCAAGTTGTTTTGCTAAGGACAGTGTTTTTGCTCTCTCTGCAGGAG
TTTTCATCTTCTCCTTCTTAAAAATATCAGTTATGGACATTTGTTTTGCAGCATCTGGCACAATACGTTACTTCTCTCCT
TTGTTCTGAGGGTCAGGTTTCATTAAGCAGTTTCCTCTTTTTGCTTTGATGACGAGGAGGAGCATAAAATCATGAGGTTG
AGTATCTCTCAGTGGAAATTTAGTTCTACCGTTTATTTTGGAGACACTTAGGGGATATCAACCAGAAAAAGCCAACTTTT
TCCTCCACCAGAACCACTTCTTGCTAGCGACACAGGTAACAATAACTGTTTTTATTTGTTTTCTACTAAACTATCAAATA
TATTTTCAGGTAGTGTTATCTGAGCGCTCGATTGATGGGGAAGGCCTGTTAGTTGTTTTAGATCAGGTTAAATCCGGGAG
CATGTGACAACTGTGATTCTTCATCAATTGTATTCTTTAGGAGTATGCTGTGATGTGTGTTGTGTGCTAAGAAATTAAGG
TAGAGGGACCTATGGAGATCATTATTCTTTAACAGGGTTAAATGTTTATATATTACCCACCTAAATGATGCCATAATAGG
ATACAGGTAAAAAAGGATCAGTGTGTAAATGAACCTGATACACAAGGGTGACTGACCACCTAGTTTGATCCCTGGACTTC
ACATGAAGGTGGAGTGGGAGAACTCACCTTGTAAGATTGCCTTCTGGACACGTGCACCTTGGCCCAACTATCCCAAGAGC
AGTAATAATAAATAACAATTTTGAAAAAGAATTTGGTAATGGAAGGATTCTTTTTTGTCAAGATAAAAGGCAAAAGGTGA
ATCACTTCAGAAAATTTCATAAATTAGAAAAATTTGAGAAGAATGCTCTCATATATGCAACTTGTATTTATTATCTGCTG
GATCTCAGCTCGGTTATTAATGTAAAGGTAATGTCAGTTACAAATATTTACTTATTATTTACAAGTACTAACTATTTACA
AATACAGTGTTATCTACTATCAGATGGTTTCCTTCATGGACATTAGCTTATGTGTACTGCATTGTGACAAAATTTATTTT
CATATAGCCTCCCCTTGCAACATCATGTGTGTATATTTTATATGTGACATGATCATGAACATGAAATGCTGTGAGATTAA
AAGTTCATAATGTATCCTCTAAGACTCTCCCTGTTGCTCTCACACCCCATCCAGCAATGATCCCTTTCTTCTTCTCAGTT
TGCTGTCGGTACTAAAGGATAGAAATCATTTCTGCTGACTTTGAGCTGTGAGAATCAAATGATATAGAATATATCACATT
TTTTTTCTGGGTATGGTTTAACCCACTCATTAGGAACTTTGCTGGTTGAATCCACTTACCTGGTTGAAGCATGGAGATGA
ATGTATAGAAAGTGGCTGGGTGGAAAGTTTGGATATCTTAGCTGATTAGGGCTTCAGTACAACAAATATTTACGTATGTT
GGATCACCTTCAGTATTAACCTTCAAATACCTTTCGTAAGGTACTGTCAGGGAAACTCTGACAGTCATGTATAAATAAAA
AGACAGAAAGAACAATTACTATTCATTTATTATCAAAATGTATAAATAATTTTAAAGCAACATTTTTATTTACTCCTAAA
ATTTCAAAGAGGCTATAACAATTATAGAATGACTTAGGAAATTTTTAATCTTTCCATATTTCTTAGAAATAAGTGGGTGG
GTATGGGGGACTTTTGGTATAGCATTGGAAATGTAAATGAGCTGAGCTAAATACCTAATAAAAAATGGGAAAAAAAAGAA
ATAAGTGCTGTGACCATTAGAAATATAAACTACTTTAATAAAAATCTACAATGAGCTCTCACTTGTAATGTAAATTGGGT
CAGTCCATGTCAGTGTGTTTGTAGCATTAAGTGAGTTAGTCAGACCCCACCTTTTTCCAGCCCTCTGTATCATCAGATTC
AGTTCTCTTCCTATAGGTGAACATTTTAATATTTTTCTTAGAAACCACTCGAGGCACCaagctt

A-429+97

gatatcGCTGCAACTAGCATAATTCAAAATGAAGAAATAACTTAAAAATTAGTAACATAATAGTAAATTTCCAACAGTCT
ATTACAAAACTAGGAGCATCAGTGCCACATTTTTCCAAGTTGTTTTGCTAAGGACAGTGTTTTTGCTCTCTCTGCAGGAG
TTTTCATCTTCTCCTTCTTAAAAATATCAGTTATGGACATTTGTTTTGCAGCATCTGGCACAATACGTTACTTCTCTCCT
TTGTTCTGAGGGTCAGGTTTCATTAAGCAGTTTCCTCTTTTTGCTTTGATGACGAGGAGGAGCATAAAATCATGAGGTTG
AGTATCTCTCAGTGGAAATTTAGTTCTACCGTTTATTTTGGAGACACTTAGGGGATATCAACCAGAAAAAGCCAACTTTT
TCCTCCACCAGAACCACTTCTTGCTAGCGACACAGGTAACAATAACTGTTTTTATTTGTTTTCTACTAAACTATCAAATA
TATTTTCAGGTAGTGTTATCTGAGCGCTCGATTGATGGGGAAGGCCTGTTAGTTGTTTTAGATCAGGTTAAATCCGGGAG
CATGTGACAACTGTGATTCTTCATCAATTGTATTCTTTAGGAGTATGCTGTGATGTGTGTTGTGTGCTAAGAAATTAAGG
TAGAGGGACCTATGGAGATCATTATTCTTTAACAGGGTTAAATGTTTATATATTACCCACCTAAATGATGCCATAATAGG
ATACAGGTAAAAAAGGATCAGTGTGTAAATGAACCTGATACACAAGGGTGACTGACCACCTAGTTTGATCCCTGGACTTC
ACATGAAGGTGGAGTGGGAGAACTCACCTTGTAAGATTGCCTTCTGGACACGTGCACCTTGGCCCAACTATCCCAAGAGC
AGTAATAATAAATAACAATTTTGAAAAAGAATTTGGTAATGGAAGGATTCTTTTTTGTCAAGATAAAAGGCAAAAGGTGA
ATCACTTCAGAAAATTTCATAAATTAGAAAAATTTGAGAAGAATGCTCTCATATATGCAACTTGTATTTATTATCTGCTG
GATCTCAGCTCGGTTATTAATGTAAAGGTAATGTCAGTTACAAATATTTACTTATTATTTACAAGTACTAACTATTTACA
AATACAGTGTTATCTACTATCAGATGGTTTCCTTCATGGACATTAGCTTATGTGTACTGCATTGTGACAAAATTTATTTT
CATATAGCCTCCCCTTGCAACATCATGTGTGTATATTTTATATGTGACATGATCATGAACATGAAATGCTGTGAGATTAA
AAGTTCATAATGTATCCTCTAAGACTCTCCCTGTTGCTCTCACACCCCATCCAGCAATGATCCCTTTCTTCTTCTCAGTT
TGCTGTCGGTACTAAAGGATAGAAATCATTTCTGCTGACTTTGAGCTGTGAGAATCAAATGATATAGAATATATCACATT
TTTTTTCTGGGTATGGTTTAACCCACTCATTAGGAACTTTGCTGGTTGAATCCACTTACCTGGTTGAAGCATGGAGATGA
ATGTATAGAAAGTGGCTGGGTGGAAAGTTTGGATATCTTAGCTGATTAGGGCTTCAGTACAACAAATATTTACGTATGTT
GGATCACCTTCAGTATTAACCTTCAAATACCTTTCGTAAGGTACTGTCAGGGAAACTCTGACAGTCATGTATAAATAAAA
AGACAGAAAGAACAATTACTATTCATTTATTATCAAAATGTATAAATAATTTTAAAGCAACATTTTTATTTACTCCTAAA
ATTTCAAAGAGGCTATAACAATTATAGAATGACTTAGGAAATTTTTAATCTTTCCATATTTCTTAGAAATAAGTGGGTGG
GTATGGGGGACTTTTGGTATAGCATTGGAAATGTAAATGAGCTGAGCTAAATACCTAATAAAAAATGGGAAAAAAAAGAA
ATAAGTGCTGTGACCATTAGAAATATAAACTACTTTAATAAAAATCTACAATGAGCTCTCACTTGTAATGTAAATTGGGT
CAGTCCATGTCAGTGTGTTTGTAGCATTAAGTGAGTTAGTCAGACCCCACCTTTTTCCAGCCCTCTGTATCATCAGATTC
AGTTCTCTTCCTATAGGTGAACATTTTAATATTTTTCTTAGAAACCACTCGAGGCACCATTTGAACTGAGAACATACTTT
ATATATCAATCCCAAGATGAGTGAGCAGGAGGTCACTTATTCAATGGTGAGATTTCATAAATCTGCAGGATTGCAGAAAC
AAGTGAGACCTGAGGAGACTAAAGGGCCCAGAGAAGaagctt

A-429+98

gatatcGCTGCAACTAGCATAATTCAAAATGAAGAAATAACTTAAAAATTAGTAACATAATAGTAAATTTCCAACAGTCT
ATTACAAAACTAGGAGCATCAGTGCCACATTTTTCCAAGTTGTTTTGCTAAGGACAGTGTTTTTGCTCTCTCTGCAGGAG
TTTTCATCTTCTCCTTCTTAAAAATATCAGTTATGGACATTTGTTTTGCAGCATCTGGCACAATACGTTACTTCTCTCCT
TTGTTCTGAGGGTCAGGTTTCATTAAGCAGTTTCCTCTTTTTGCTTTGATGACGAGGAGGAGCATAAAATCATGAGGTTG
AGTATCTCTCAGTGGAAATTTAGTTCTACCGTTTATTTTGGAGACACTTAGGGGATATCAACCAGAAAAAGCCAACTTTT
TCCTCCACCAGAACCACTTCTTGCTAGCGACACAGGTAACAATAACTGTTTTTATTTGTTTTCTACTAAACTATCAAATA
TATTTTCAGGTAGTGTTATCTGAGCGCTCGATTGATGGGGAAGGCCTGTTAGTTGTTTTAGATCAGGTTAAATCCGGGAG
CATGTGACAACTGTGATTCTTCATCAATTGTATTCTTTAGGAGTATGCTGTGATGTGTGTTGTGTGCTAAGAAATTAAGG
TAGAGGGACCTATGGAGATCATTATTCTTTAACAGGGTTAAATGTTTATATATTACCCACCTAAATGATGCCATAATAGG
ATACAGGTAAAAAAGGATCAGTGTGTAAATGAACCTGATACACAAGGGTGACTGACCACCTAGTTTGATCCCTGGACTTC
ACATGAAGGTGGAGTGGGAGAACTCACCTTGTAAGATTGCCTTCTGGACACGTGCACCTTGGCCCAACTATCCCAAGAGC
AGTAATAATAAATAACAATTTTGAAAAAGAATTTGGTAATGGAAGGATTCTTTTTTGTCAAGATAAAAGGCAAAAGGTGA
ATCACTTCAGAAAATTTCATAAATTAGAAAAATTTGAGAAGAATGCTCTCATATATGCAACTTGTATTTATTATCTGCTG
GATCTCAGCTCGGTTATTAATGTAAAGGTAATGTCAGTTACAAATATTTACTTATTATTTACAAGTACTAACTATTTACA
AATACAGTGTTATCTACTATCAGATGGTTTCCTTCATGGACATTAGCTTATGTGTACTGCATTGTGACAAAATTTATTTT
CATATAGCCTCCCCTTGCAACATCATGTGTGTATATTTTATATGTGACATGATCATGAACATGAAATGCTGTGAGATTAA
AAGTTCATAATGTATCCTCTAAGACTCTCCCTGTTGCTCTCACACCCCATCCAGCAATGATCCCTTTCTTCTTCTCAGTT
TGCTGTCGGTACTAAAGGATAGAAATCATTTCTGCTGACTTTGAGCTGTGAGAATCAAATGATATAGAATATATCACATT
TTTTTTCTGGGTATGGTTTAACCCACTCATTAGGAACTTTGCTGGTTGAATCCACTTACCTGGTTGAAGCATGGAGATGA
ATGTATAGAAAGTGGCTGGGTGGAAAGTTTGGATATCTTAGCTGATTAGGGCTTCAGTACAACAAATATTTACGTATGTT
GGATCACCTTCAGTATTAACCTTCAAATACCTTTCGTAAGGTACTGTCAGGGAAACTCTGACAGTCATGTATAAATAAAA
AGACAGAAAGAACAATTACTATTCATTTATTATCAAAATGTATAAATAATTTTAAAGCAACATTTTTATTTACTCCTAAA
ATTTCAAAGAGGCTATAACAATTATAGAATGACTTAGGAAATTTTTAATCTTTCCATATTTCTTAGAAATAAGTGGGTGG
GTATGGGGGACTTTTGGTATAGCATTGGAAATGTAAATGAGCTGAGCTAAATACCTAATAAAAAATGGGAAAAAAAAGAA
ATAAGTGCTGTGACCATTAGAAATATAAACTACTTTAATAAAAATCTACAATGAGCTCTCACTTGTAATGTAAATTGGGT
CAGTCCATGTCAGTGTGTTTGTAGCATTAAGTGAGTTAGTCAGACCCCACCTTTTTCCAGCCCTCTGTATCATCAGATTC
AGTTCTCTTCCTATAGGTGAACATTTTAATATTTTTCTTAGAAACCACTCGAGGCACCATTTGAACTGAGAACATACTTT
ATATATCAATCCCAAGATGAGTGAGCAGGAGGTCACTTATTCAATGGTGAGATTTCATAAATCTGCAGGATTGCAGAAAC
AAGTGAGACCTGAGGAGACTAAAGGGCCCAGAGAAGCaagctt

G-462-39

gatatcGCTGCAATTAGAATAATTCAAAATGAAGAAATAACTTAAAAATTAGAAACATAATAGTAAATTTCCAAAATTAC
AAATTACAAATTATAATTTAAATTACAAATTATAATTACAAAACAAGGAGCATCAGTCCCACATTTTTCCTAGTTGTTTT
GCTAAGGATAGTGTTTTTGCTCTCTCTGCAGGAGTGTTCATCTTCTCTGTCTTAAAAATATCAGTTATGGACATTTGTTT
TGCAGCATCTGGCACAATATGTTTCTTCTCTCCTTTGTTCTGAGGGTCAGGTTTCATTAAGCAGTTTCCTCTTTTTGCTT
TGATGAAGAGGAGGAGCATAAAATCATGAGGTTGAGTATCACTCAGTGGAAATTTAGTTCTACTGTTTATTTTGGAGACA

CTTAGGGGATATCAACCAGAAAAAGCCAACTTTTTCTCCACAGGAATCACTTCTCAGTAGAGACACAGGTAACAATAACT
GCTTTTATTGGTTTTCTACTAAATGATCAAATATAATTTCAGATAGTGTTATGTGAGCGCTCGATTGATGGGGAAGGCCT
GTTAGTTGTTTTAGATCAGGTTAAATCTGGCAGCATGTGACAACTGTGACTCTGCATTAATTTTATTCTTTAGGAGTATG
CTGTGATGTGTGTTGTGTGCTAAGAAATTAAGGTAGAGGGACCTATGGAGTTCATTATTCTTTAACTGGGTTAAATGTTT
ATATATTACCCACCTAAATGATGTCATAATAGTTTGCAGGGAAAAAGGGTCAGTGTGTAAATGAACTTGCTACACAAGGA
TAACTGACCACCTAGTTTAATCCGTGGACTTCACATGAAGGTGGAGTGGGAGAACTCACTTTGTAAAAACGCCTTCTGGA
CACCTTGGCTCAACTATCCCAAGAGCAATAATAATAAATAACAATTTTGAAAAAGAATTGGTAATGGAAAGATTCTTTTT
TGTCAAGATAAAAGGCAAAAGGTGAATCACTTCAGAAAATTTCATAAATTAGAAAAACTTGAGAAGAATGCTCTCACATG
TGAGACTTGTATTTATTATCTGCTGGATCTCAGCTCGGTTATTAATGTAAAGATAATGTCTGTTACAAATACTTACTATT
TACATGTACTAACTATTTACAAATACAGTGTTATCTACTATCAGATGGTTTCCTTCATGGACATTAGCTTCTGTATACTG
CATTGTGACAAAATTTATTTTCATATAGCCTCCCCTTGTAACATCATGTGTGTTTATATTATATGTGACATGATCATGAA
CATGAAATGCTGTGAGATTAAAAGTTCATAATGTATCCTCTATGCCTCTCTCTGTTCCTCCCACACCCCATTCAGCAATG
ACCCCTTTCTTCTTCTCTGTTTGCTGTCGGTACTAAAGGATAGACATCATTTCTGCTGACTTTGAGCTATGGGACTCAGA
TGATATAGAATATACCACATTTTTCTTTCTGGGTCTGGTTTATCCCACTCATTAGGAACTTTTCTTGTTGAATCTACTTA
CCCGGTTGAAGCATGGAGTAGAGTCTAGAGAATGTGGCTGGGTGGAGAGGTTAGACATTAGCTCATTGGGGCTTCAATCC
AAGAGATATTTATGTATATTGGATCACCTGTAGTATTAACCTTCAAATACCTTTCTTAAGGTAGTGTCAGGGAAACTCTG
ACAGTCATGTATAAATAAAAAGACGGAAAAACAATTAATATTCATTTATTATCAAAATGCTTAAATAATTTTAAAGCAAC
ATTTTTATTTATTCCTAAAATTTCAAAGAGATTATATCAATTGTAGAATGACTTATTAAATTTTTAATTCTTTCCATATT
TCTTAGAAATAATTGCTGTGGCCATTATAAACGACTTTAACAAAAATCTACAATGAGTTCTCACCTGTAACGGAAATTTG
GTCAGTCCATGTCAGGGTGTTTATAGCATTAAGTGAGTTAGTCAGACCCCACCCTTTCCCAGACCTCTGTATCATCATAT
CTAGTTATCTTCCTATAGGTGAACATTTTAACATTTTTCGTAGAAACCACTCAAGGCACCaagctt

G-462+97

gatatcGCTGCAATTAGAATAATTCAAAATGAAGAAATAACTTAAAAATTAGAAACATAATAGTAAATTTCCAAAATTAC
AAATTACAAATTATAATTTAAATTACAAATTATAATTACAAAACAAGGAGCATCAGTCCCACATTTTTCCTAGTTGTTTT
GCTAAGGATAGTGTTTTTGCTCTCTCTGCAGGAGTGTTCATCTTCTCTGTCTTAAAAATATCAGTTATGGACATTTGTTT
TGCAGCATCTGGCACAATATGTTTCTTCTCTCCTTTGTTCTGAGGGTCAGGTTTCATTAAGCAGTTTCCTCTTTTTGCTT
TGATGAAGAGGAGGAGCATAAAATCATGAGGTTGAGTATCACTCAGTGGAAATTTAGTTCTACTGTTTATTTTGGAGACA
CTTAGGGGATATCAACCAGAAAAAGCCAACTTTTTCTCCACAGGAATCACTTCTCAGTAGAGACACAGGTAACAATAACT
GCTTTTATTGGTTTTCTACTAAATGATCAAATATAATTTCAGATAGTGTTATGTGAGCGCTCGATTGATGGGGAAGGCCT
GTTAGTTGTTTTAGATCAGGTTAAATCTGGCAGCATGTGACAACTGTGACTCTGCATTAATTTTATTCTTTAGGAGTATG
CTGTGATGTGTGTTGTGTGCTAAGAAATTAAGGTAGAGGGACCTATGGAGTTCATTATTCTTTAACTGGGTTAAATGTTT
ATATATTACCCACCTAAATGATGTCATAATAGTTTGCAGGGAAAAAGGGTCAGTGTGTAAATGAACTTGCTACACAAGGA
TAACTGACCACCTAGTTTAATCCGTGGACTTCACATGAAGGTGGAGTGGGAGAACTCACTTTGTAAAAACGCCTTCTGGA
CACCTTGGCTCAACTATCCCAAGAGCAATAATAATAAATAACAATTTTGAAAAAGAATTGGTAATGGAAAGATTCTTTTT
TGTCAAGATAAAAGGCAAAAGGTGAATCACTTCAGAAAATTTCATAAATTAGAAAAACTTGAGAAGAATGCTCTCACATG
TGAGACTTGTATTTATTATCTGCTGGATCTCAGCTCGGTTATTAATGTAAAGATAATGTCTGTTACAAATACTTACTATT
TACATGTACTAACTATTTACAAATACAGTGTTATCTACTATCAGATGGTTTCCTTCATGGACATTAGCTTCTGTATACTG
CATTGTGACAAAATTTATTTTCATATAGCCTCCCCTTGTAACATCATGTGTGTTTATATTATATGTGACATGATCATGAA
CATGAAATGCTGTGAGATTAAAAGTTCATAATGTATCCTCTATGCCTCTCTCTGTTCCTCCCACACCCCATTCAGCAATG
ACCCCTTTCTTCTTCTCTGTTTGCTGTCGGTACTAAAGGATAGACATCATTTCTGCTGACTTTGAGCTATGGGACTCAGA
TGATATAGAATATACCACATTTTTCTTTCTGGGTCTGGTTTATCCCACTCATTAGGAACTTTTCTTGTTGAATCTACTTA
CCCGGTTGAAGCATGGAGTAGAGTCTAGAGAATGTGGCTGGGTGGAGAGGTTAGACATTAGCTCATTGGGGCTTCAATCC
AAGAGATATTTATGTATATTGGATCACCTGTAGTATTAACCTTCAAATACCTTTCTTAAGGTAGTGTCAGGGAAACTCTG
ACAGTCATGTATAAATAAAAAGACGGAAAAACAATTAATATTCATTTATTATCAAAATGCTTAAATAATTTTAAAGCAAC
ATTTTTATTTATTCCTAAAATTTCAAAGAGATTATATCAATTGTAGAATGACTTATTAAATTTTTAATTCTTTCCATATT
TCTTAGAAATAATTGCTGTGGCCATTATAAACGACTTTAACAAAAATCTACAATGAGTTCTCACCTGTAACGGAAATTTG
GTCAGTCCATGTCAGGGTGTTTATAGCATTAAGTGAGTTAGTCAGACCCCACCCTTTCCCAGACCTCTGTATCATCATAT
CTAGTTATCTTCCTATAGGTGAACATTTTAACATTTTTCGTAGAAACCACTCAAGGCACCATTTTAACTGAGAACATACT
TCATACATCATTCCCAAGATGAGTGAGCAGGAGGTCACTTACTCAACTGTGAGATTTCATGAGTCTTCAAGGTTGCAGAA
ACTAGTGAGGACTGAGGAGCCTCAAAGGCCCAGAGAAGaagctt

G-462+98

gatatcGCTGCAATTAGAATAATTCAAAATGAAGAAATAACTTAAAAATTAGAAACATAATAGTAAATTTCCAAAATTAC
AAATTACAAATTATAATTTAAATTACAAATTATAATTACAAAACAAGGAGCATCAGTCCCACATTTTTCCTAGTTGTTTT
GCTAAGGATAGTGTTTTTGCTCTCTCTGCAGGAGTGTTCATCTTCTCTGTCTTAAAAATATCAGTTATGGACATTTGTTT
TGCAGCATCTGGCACAATATGTTTCTTCTCTCCTTTGTTCTGAGGGTCAGGTTTCATTAAGCAGTTTCCTCTTTTTGCTT
TGATGAAGAGGAGGAGCATAAAATCATGAGGTTGAGTATCACTCAGTGGAAATTTAGTTCTACTGTTTATTTTGGAGACA
CTTAGGGGATATCAACCAGAAAAAGCCAACTTTTTCTCCACAGGAATCACTTCTCAGTAGAGACACAGGTAACAATAACT
GCTTTTATTGGTTTTCTACTAAATGATCAAATATAATTTCAGATAGTGTTATGTGAGCGCTCGATTGATGGGGAAGGCCT
GTTAGTTGTTTTAGATCAGGTTAAATCTGGCAGCATGTGACAACTGTGACTCTGCATTAATTTTATTCTTTAGGAGTATG
CTGTGATGTGTGTTGTGTGCTAAGAAATTAAGGTAGAGGGACCTATGGAGTTCATTATTCTTTAACTGGGTTAAATGTTT
ATATATTACCCACCTAAATGATGTCATAATAGTTTGCAGGGAAAAAGGGTCAGTGTGTAAATGAACTTGCTACACAAGGA
TAACTGACCACCTAGTTTAATCCGTGGACTTCACATGAAGGTGGAGTGGGAGAACTCACTTTGTAAAAACGCCTTCTGGA
CACCTTGGCTCAACTATCCCAAGAGCAATAATAATAAATAACAATTTTGAAAAAGAATTGGTAATGGAAAGATTCTTTTT
TGTCAAGATAAAAGGCAAAAGGTGAATCACTTCAGAAAATTTCATAAATTAGAAAAACTTGAGAAGAATGCTCTCACATG
TGAGACTTGTATTTATTATCTGCTGGATCTCAGCTCGGTTATTAATGTAAAGATAATGTCTGTTACAAATACTTACTATT
TACATGTACTAACTATTTACAAATACAGTGTTATCTACTATCAGATGGTTTCCTTCATGGACATTAGCTTCTGTATACTG

CATTGTGACAAAATTTATTTTCATATAGCCTCCCCTTGTAACATCATGTGTGTTTATATTATATGTGACATGATCATGAA
CATGAAATGCTGTGAGATTAAAAGTTCATAATGTATCCTCTATGCCTCTCTCTGTTCCTCCCACACCCCATTCAGCAATG
ACCCCTTTCTTCTTCTCTGTTTGCTGTCGGTACTAAAGGATAGACATCATTTCTGCTGACTTTGAGCTATGGGACTCAGA
TGATATAGAATATACCACATTTTTCTTTCTGGGTCTGGTTTATCCCACTCATTAGGAACTTTTCTTGTTGAATCTACTTA
CCCGGTTGAAGCATGGAGTAGAGTCTAGAGAATGTGGCTGGGTGGAGAGGTTAGACATTAGCTCATTGGGGCTTCAATCC
AAGAGATATTTATGTATATTGGATCACCTGTAGTATTAACCTTCAAATACCTTTCTTAAGGTAGTGTCAGGGAAACTCTG
ACAGTCATGTATAAATAAAAAGACGGAAAAACAATTAATATTCATTTATTATCAAAATGCTTAAATAATTTTAAAGCAAC
ATTTTTATTTATTCCTAAAATTTCAAAGAGATTATATCAATTGTAGAATGACTTATTAAATTTTTAATTCTTTCCATATT
TCTTAGAAATAATTGCTGTGGCCATTATAAACGACTTTAACAAAAATCTACAATGAGTTCTCACCTGTAACGGAAATTTG
GTCAGTCCATGTCAGGGTGTTTATAGCATTAAGTGAGTTAGTCAGACCCCACCCTTTCCCAGACCTCTGTATCATCATAT
CTAGTTATCTTCCTATAGGTGAACATTTTAACATTTTTCGTAGAAACCACTCAAGGCACCATTTTAACTGAGAACATACT
TCATACATCATTCCCAAGATGAGTGAGCAGGAGGTCACTTACTCAACTGTGAGATTTCATGAGTCTTCAAGGTTGCAGAA
ACTAGTGAGGACTGAGGAGCCTCAAAGGCCCAGAGAAGCaagctt

Ly49B fragments

[Exon 1 highlighted in blue, unique sequence in grey, LINE sequence in green]

B-1513-12

gatatcGGTACTACCAGAGGATCCCTCAATACCTCTGCTGGGCATACATCCAGAAGATGTTCCAATTAGTAAGAAAGAAA
TATGCTCCACTATGTTCATAGTAGCCTTATTTATAATAGCCAGAAGCTGGAAAGAACCCAGATGCCCCTCAACAGAGGAA
TGGATACAAAAATTGTGGTACATTTATACAATGGAGTACTACTCAGCTATTAAAAAGAATGGATTTATGAAATTCCTAGG
CAAATGGTTGGACTTGGAGGGCATTATGCTGAGTGAGGTAACCCAATCACAAAAGAACTCAAATGATATGTACTCACTGA
TAAGTGGATATTAGCCCAGAAACTTAGAATACCCAAGATATAAGTTACAATTTGCAAAACACATGAAACTCAAGAAGAAC
GAAGACCTAAGTATGGACACCTTGCCCCTTCTTAGAATTGGGAATAAAACACCCATGGAAGGAGTTACAGAGACAAAGTT
TGGAGCTGAGACAAAAGGATGGACCATCTAGAGACTGCCATATCCAGGGATCCATCCCATAATCAGCCTCCAAACGCTGA
CACCATTTCATACACTAGCAAGATTTTGCTGATAGGACCCTGATATAGCTGTCTCTTGTGAGACTACGCAGAGGCCTAGC
AAACACAGAAGTGGATGCTCACAGTCAGCTATTGGATGGATCACAGGGCCCCCAATAAAGGAGCTAGAGAAAGTACCCAA
GGAGCTAAAGGGATGTGCAACCCTATAGATGGAACAACATTATGAACTAACCAGTACCCCGGAGCTTTTGACTCTAGCTG
CATATGTATCAAAAGATGGCCTAGCCAGCCATCATTGTAAACATAGGCCCATTGGACTTGCAAACTTTTTATGCAGGGGA
ATGAATGGGCCAAAAATGGGAGTGGGTGGGTAGGGGAGTTGGGGGGAGGGTATGGGGGACTTTTGGGATAACTTTGGAAA
TGTAAATGAGGAAAATACCTAACTAAAAATAAAAAAAAACTTAGTTTAACAGTTAAAAAAAAAGAACTTTAACAGTGCAA
ATGCCTTTAAAAGGTATTCGTAAAACACACAGGTACAATAAGTACAATTCAACAACAAGTAAACATTTTCAAATAGAAAA
AGAAAATTTCCAACAGCCTATTACAAAATTAGAAGCAGGGGAATTTCAGTCCCCCATTCTTTTCCTATGGTTTTGTTAAG
GACACTTATTTTTCTTTCTCTGAAGGAGTGGTATCTTTTCAGTATTCAACTATCAATTATGGAAATCTATTGCAGTATAT
GCCAGAATACATTGGTCCTTTCCTTTGCTGTCAGAGTCAGCTTTTATTATGCAATTTCCTCTTTTTGCTTAGATCAAGAG
GAGGGGCCGAATGTTATGAGCTGGAGTATTACTCAGAGATGATTCAGTTCCGTCATTCTTCTTGAAGCTCTGAGTGTATA
CAGACCAGGAATGGCCAACTTGTCCCTCAGTTAGTGGATTAATTCCCTTCACCAGAATTACTTCCTGGaagctt

B-660-12

gatatcGCAAACTTTTTATGCAGGGGAATGAATGGGCCAAAAATGGGAGTGGGTGGGTAGGGGAGTTGGGGGGAGGGTAT
GGGGGACTTTTGGGATAACTTTGGAAATGTAAATGAGGAAAATACCTAACTAAAAATAAAAAAAAACTTAGTTTAACAGT
TAAAAAAAAAGAACTTTAACAGTGCAAATGCCTTTAAAAGGTATTCGTAAAACACACAGGTACAATAAGTACAATTCAAC
AACAAGTAAACATTTTCAAATAGAAAAAGAAAATTTCCAACAGCCTATTACAAAATTAGAAGCAGGGGAATTTCAGTCCC
CCATTCTTTTCCTATGGTTTTGTTAAGGACACTTATTTTTCTTTCTCTGAAGGAGTGGTATCTTTTCAGTATTCAACTAT
CAATTATGGAAATCTATTGCAGTATATGCCAGAATACATTGGTCCTTTCCTTTGCTGTCAGAGTCAGCTTTTATTATGCA
ATTTCCTCTTTTTGCTTAGATCAAGAGGAGGGGCCGAATGTTATGAGCTGGAGTATTACTCAGAGATGATTCAGTTCCGT
CATTCTTCTTGAAGCTCTGAGTGTATACAGACCAGGAATGGCCAACTTGTCCCTCAGTTAGTGGATTAATTCCCTTCACC
AGAATTACTTCCTGGaagctt

B-647-12

gatatcGCAGGGGAATGAATGGGCCAAAAATGGGAGtGGGTGGGTAGGGGAGTTGGGGGGAGGGTATGGGGGACTTTTGG
GATAACTTTGGAAATGTAAATGAGGAAAATACCTAACTAAAAATAAAAAAAAACTTAGTTTAACAGTTAAAAAAAAAGAA
CTTTAACAGTGCAAATGCCTTTAAAAGGTATTCGTAAAACACACAGGTACAATAAGTACAATTCAACAACAAGTAAACAT
TTTCAAATAGAAAAAGAAAATTTCCAACAGCCTATTACAAAATTAGAAGCAGGGGAATTTCAGTCCCCCATTCTTTTCCT
ATGGTTTTGTTAAGGACACTTATTTTTCTTTCTCTGAAGGAGTGGTATCTTTTCAGTATTCAACTATCAATTATGGAAAT
CTATTGCAGTATATGCCAGAATACATTGGTCCTTTCCTTTGCTGTCAGAGTCAGCTTTTATTATGCAATTTCCTCTTTTT
GCTTAGATCAAGAGGAGGGGCCGAATGTTATGAGCTGGAGTATTACTCAGAGATGATTCAGTTCCGTCATTCTTCTTGAA
GCTCTGAGTGTATACAGACCAGGAATGGCCAACTTGTCCCTCAGTTAGTGGATTAATTCCCTTCACCAGAATTACTTCCT
GGaagctt

B-520-12

gatatcTTAGTTTAACAGTTAAAAAAAAAGAACTTTAACAGTGCAAATGCCTTTAAAAGGTATTCGTAAAACACACAGGT
ACAATAAGTACAATTCAACAACAAGTAAACATTTTCAAATAGAAAAAGAAAATTTCCAACAGCCTATTACAAAATTAGAA
GCAGGGGAATTTCAGTCCCCCATTCTTTTCCTATGGTTTTGTTAAGGACACTTATTTTTCTTTCTCTGAAGGAGTGGTAT
CTTTTCAGTATTCAACTATCAATTATGGAAATCTATTGCAGTATATGCCAGAATACATTGGTCCTTTCCTTTGCTGTCAG
AGTCAGCTTTTATTATGCAATTTCCTCTTTTTGCTTAGATCAAGAGGAGGGGCCGAATGTTATGAGCTGGAGTATTACTC
AGAGATGATTCAGTTCCGTCATTCTTCTTGAAGCTCTGAGTGTATACAGACCAGGAATGGCCAACTTGTCCCTCAGTTAG
TGGATTAATTCCCTTCACCAGAATTACTTCCtGGaagctt

B-485-12
gatatcGTGCAAATGCCTTTAAAAGGTATTCGTAAAACACACAGGTACAATAAGTACAATTCAACAACAAGTAAACATTT
TCAAATAGAAAAAGAAAATTTCCAACAGCCTATTACAAAATTAGAAGCAGGGGAATTTCAGTCCCCCATTCTTTTCCTAT
GGTTTTGTTAAGGACACTTATTTTTCTTTCTCTGAAGGAGTGGTATCTTTTCAGTATTCAACTATCAATTATGGAAATCT
ATTGCAGTATATGCCAGAATACATTGGTCCTTTCCTTTGCTGTCAGAGTCAGCTTTTATTATGCAATTTCCTCTTTTTGC
TTAGATCAAGAGGAGGGGCCGAATGTTATGAGCTGGAGTATTACTCAGAGATGATTCAGTTCCGTCATTCTTCTTGAAGC
TCTGAGTGTATACAGACCAGGAATGGCCAACTTGTCCCTCAGTTAGTGGATTAATTCCCTTCACCAGAATTACTTCCTGG
aagctt

B-448-12
gatatcGGTACAATAAGTACAATTCAACAACAAGTAAACATTTTCAAATAGAAAAAGAAAATTTCCAACAGCCTATTACA
AAATTAGAAGCAGGGGAATTTCAGTCCCCCATTCTTTTCCTATGGTTTTGTTAAGGACACTTATTTTTCTTTCTCTGAAG
GAGTGGTATCTTTTCAGTATTCAACTATCAATTATGGAAATCTATTGCAGTATATGCCAGAATACATTGGTCCTTTCCTT
TGCTGTCAGAGTCAGCTTTTATTATGCAATTTCCTCTTTTTGCTTAGATCAAGAGGAGGGGCCGAATGTTATGAGCTGGA
GTATTACTCAGAGATGATTCAGTTCCGTCATTCTTCTTGAAGCTCTGAGTGTATACAGACCAGGAATGGCCAACTTGTCC
CTCAGTTAGTGGATTAATTCCCTTCACCAGAATTACTTCCTGGaagctt

B-299-12
gatatcTGAAGGAGTGGTATCTTTTCAGTATTCAACTATCAATTATGGAAATCTATTGCAGTATATGCCAGAATACATTG
GTCCTTTCCTTTGCTGTCAGAGTCAGCTTTTATTATGCAATTTCCTCTTTTTGCTTAGATCAAGAGGAGGGGCCGAATGT
TATGAGCTGGAGTATTACTCAGAGATGATTCAGTTCCGTCATTCTTCTTGAAGCTCTGAGTGTATACAGACCAGGAATGG
CCAACTTGTCCCTCAGTTAGTGGATTAATTCCCTTCACCAGAATTACTTCCTGGaagctt

B-299-95
gatatcTGAAGGAGTGGTATCTTTTCAGTATTCAACTATCAATTATGGAAATCTATTGCAGTATATGCCAGAATACATTG
GTCCTTTCCTTTGCTGTCAGAGTCAGCTTTTATTATGCAATTTCCTCTTTTTGCTTAGATCAAGAGGAGGGGCCGAATGT
TATGAGCTGGAGTATTACTCAGAGATGATTCAGTTCCGTCATTCTTCTTGAaagctt

B-299-110
gatatcTGAAGGAGTGGTATCTTTTCAGTATTCAACTATCAATTATGGAAATCTATTGCAGTATATGCCAGAATACATTG
GTCCTTTCCTTTGCTGTCAGAGTCAGCTTTTATTATGCAATTTCCTCTTTTTGCTTAGATCAAGAGGAGGGGCCGAATGT
TATGAGCTGGAGTATTACTCAGAGATGATTCAGTTCaagctt

B-299-134
gatatcTGAAGGAGTGGTATCTTTTCAGTATTCAACTATCAATTATGGAAATCTATTGCAGTATATGCCAGAATACATTG
GTCCTTTCCTTTGCTGTCAGAGTCAGCTTTTATTATGCAATTTCCTCTTTTTGCTTAGATCAAGAGGAGGGGCCGAATGT
TATGAGCTGGAGaagctt

B-299-154
gatatcTGAAGGAGTGGTATCTTTTCAGTATTCAACTATCAATTATGGAAATCTATTGAAGTATATGCCAGAATACATTG
GTCCTTTCCTTTGCTGTCAGAGTCAGCTTTTATTATGCAATTTCCTCTTTTTGCTTAGATCAAGAGGAGGGGaagctt

B-299-172
gatatcTGAAGGAGTGGTATCTTTTCAGTATTCAACTATCAATTATGGAAATCTATTGCAGTATATGCCAGAATACATTG
GTCCTTTCCTTTGCTGTCAGAGTCAGCTTTTATTATGCAATTTCCTCTTTTTGCaagctt

B-299-199
gatatcTGAAGGAGTGGTATCTTTTCAGTATTCAACTATCAATTATGGAAATCTATTGCAGTATATGCCAGAATACATTG
GTCCTTTCCTTTGCTGTCAGAGTCAGCaagctt

B-248-95
gatatcGCAGTATATGCCAGAATACATTGGTCCTTTCCTTTGCTGTCAGAGTCAGCTTTTATTATGCAATTTCCTCTTTT
TGCTTAGATCAAGAGGAGGGGCCGAATGTTATGAGCTGGAGTATTACTCAGAGATGATTCAGTTCCGTCATTCTTCTTGA
aagctt

B-213-95
gatatcGCTGTCAGAGTCAGCTTTTATTATGCAATTTCCTCTTTTTGCTTAGATCAAGAGGAGGGGCCGAATGTTATGAG
CTGGAGTATTACTCAGAGATGATTCAGTTCCGTCATTCTTCTTGAaagctt

B-182-95
gatatcCCTCTTTTTGCTTAGATCAAGAGGAGGGGCCGAATGTTATGAGCTGGAGTATTACTCAGAGATGATTCAGTTCC
GTCATTCTTCTTGAaagctt

B-152-95
CGAATGTTATGAGCTGGAGTATTACTCAGAGATGATTCAGTTCCGTCATTCTTCTTGAaagctt

B-448-Inr-GG

gatatcGGTACAATAAGTACAATTCAACAACAAGTAAACATTTTCAAATAGAAAAAGAAAATTTCCAACAGCCTATTACA
AAATTAGAAGCAGGGGAATTTCAGTCCCCCATTCTTTTCCTATGGTTTTGTTAAGGACACTTATTTTTCTTTCTCTGAAG
GAGTGGTATCTTTTCAGTATTCAACTATCAATTATGGAAATCTATTGCAGTATATGCCAGAATACATTGGTCCTTTCCTT
TGCTGTCAGAGTCAGCTTTTATTATGCAATTTCCTCTTTTTGCTTAGATCAAGAGGAGGGGCCGAATGTTATGAGCTGGA
GTATTACTCAGAGATGATTCGGTTCCGTCGTTCTTCTTGAAGCTCTGAGTGTATACAGACCAGGAATGGCCAACTTGTCC
CTCAGTTAGTGGATTAATTCCCTTCACCAGAATTACTTCCTGGaagctt

Brat-415-12

gatatcGGTACAATAAGCATAATTTAAAAACAAAAAGTTCAAATAGAAGAAAATCACAAACAGAAATTAGAAGCAGGGGA
ACTTCAGTCCTCCCCTTTTCCTAGTGGTTTTGTTACGGACACTGGTTTTGCTTGCTCTGAAGGAGTGTTATCTTCTTGGT
CTTCAGATATCAATTAAGGACATCTATTGCCGCCTATGGCATAATACATTGGCTCTTTCCTTTGAAAGTCAGCTTTCATC
ATGCAATTTCCTCTTTTTGCTCTGGCAAGAGGAGGGGCAGGAAATCATGAGCTGGCACACCACTCAGAGCTGATTCAGTT
CTATCTTTCATCTTGGAGCCCTAAGGACGAGGAAAGGCCAAATTGTTGCCCAATAGAGGATAAATTCCCTCCGCCAGAAT
CATTTCCTGGaagctt

Brat-284-12

gatatcTGAAGGAGTGTTATCTTCTTGGTCTTCAGATATCAATTAAGGACATCTATTGCCGCCTATGGCATAATACATTG
GCTCTTTCCTTTGAAAGTCAGCTTTCATCATGCAATTTCCTCTTTTTGCTCTGGCAAGAGGAGGGGCAGGAAATCATGAG
CTGGCACACCACTCAGAGCTGATTCAGTTCTATCTTTCATCTTGGAGCCCTAAGGACGAGGAAAGGCCAAATTGTTGCCC
AATAGAGGATAAATTCCCTCCGCCAGAATCATTTCCTGGaagctt
